# Delimiting genetic units in Neotropical toads under incomplete lineage sorting and hybridization

**DOI:** 10.1186/1471-2148-12-242

**Published:** 2012-12-11

**Authors:** Maria Tereza C Thomé, Kelly R Zamudio, Célio F B Haddad, João Alexandrino

**Affiliations:** 1 Departamento de Zoologia, Instituto de Biociências, UNESP - Univ Estadual Paulista, Campus Rio Claro, Caixa Postal 19913506-900, Rio Claro, SP, Brazil; 2 Department of Ecology and Evolutionary Biology, Cornell University, E209 Corson Hall, Ithaca, NY, 14853-2701, USA; 3 Departamento de Ciências Biológicas, UNIFESP - Univ Federal de São Paulo, Campus Diadema, Rua Professor Artur Riedel 275, 09972-270, Diadema, SP, Brazil

**Keywords:** *Rhinella crucifer* group, Species complex, Bufonidae, Taxonomy

## Abstract

**Background:**

Delimiting genetic units is useful to enhance taxonomic discovery and is often the first step toward understanding evolutionary mechanisms generating diversification. The six species within the *Rhinella crucifer* group of toads were defined under morphological criteria alone. Previous data suggest limited correspondence of these species to mitochondrial lineages, and morphological intergradation at transitions between forms suggests hybridization. Here we extensively sampled populations throughout the geographic distribution of the group and analyzed mitochondrial and nuclear sequence data to delimit genetic units using tree–based and allele frequency–based approaches.

**Results:**

These approaches yielded complementary results, with allele frequency-based methods performing unexpectedly well given the limited number of loci examined. Both mitochondrial and nuclear markers supported a genetic structure of five units within the group, with three of the inferred units distributed within its main range, while two other units occur in separate isolates. The inferred units are mostly discordant with currently described forms: unequivocal association exists for only two of the six species in the group. Genetic evidence for hybridization exists for two pairs of units, with clear cyto–nuclear allele mixing observed in one case.

**Conclusions:**

Our results confirmed that current taxonomy does not represent evolutionary units in the *Rhinella crucifer* group. Correspondence between genetically distinguishable units and the currently recognized species is only possible for *Rhinella henseli* and *R. inopina*. The recognition of other species relies on the reassessment of the geographic range of *R. crucifer*, the examination of the type series of *R. ornata* for hybrids, and on the use of additional markers to verify the genetic distinctiveness of *R. abei.* We state that *R. pombali* should not remain a valid species since its description appears to be based on hybrids, and that the name *R. pombali* should be considered a synonym of both *R. crucifer* and *R. ornata*. The fifth inferred but undescribed genetic unit may represent a new species. Our results underscore the potential of the *R. crucifer* species group to contribute to a better understanding of diversification processes and hybridization patterns in the Neotropics, and provide the basis for future evolutionary and taxonomic studies.

## Background

Molecular data in taxonomic studies has profoundly impacted the field by bringing fresh perspectives on discussions about species concepts and delimitation [1,2] harnessing the power of barcoding for biodiversity discovery [3,4], and leading to a more representative taxonomy [5-7]. Early efforts of genetic delimitation of Evolutionary Significant Units [8] for conservation purposes [9] met with controversy, primarily because of the criteria applied [10]. At the core of these discussions is the recognition of species as segments of population lineages against a view of species as operational units of a taxonomic rank [11]. Regardless of the adopted view, most biodiversity researchers agree that delimitation of genetic units is i) a useful proxy for enhancing the rate of taxonomic discovery and ii) the first step towards understanding evolutionary mechanisms contributing to diversification of closely related organisms, two measures that are specially relevant for conservation planning of taxa in threatened environments [12,13].

Using genetic criteria for unit delimitation has prompted a recent change of paradigm in systematics [14]. Newly available computational tools based on multilocus methods are now applied to 'species' delimitation [15], inference of the relationships among previously defined species [16-18], or both [19,20]. However, the delimitation of genetic units with tree–based methods is not straightforward because it often requires previous assignment of individuals to hypothetical species. In addition, the absence of horizontal gene flow is an assumption for most of these methods [21]. Alternatively, analyses of allele frequencies at multilocus markers can jointly explore genetic structure and infer levels of migration without *a priori* information [22], and have been used to delimit closely related species that potentially hybridize [23-27]. These methods have disadvantages over species-tree methods, such as limited phylogenetic signal, potential sensitivity to very recent genetic isolation [15], and a tradeoff between the costs of population–level sampling and the number of markers required to detect genetic structure, especially when the efficiency of the markers [28] is not known *a priori*.

**Figure 1:**
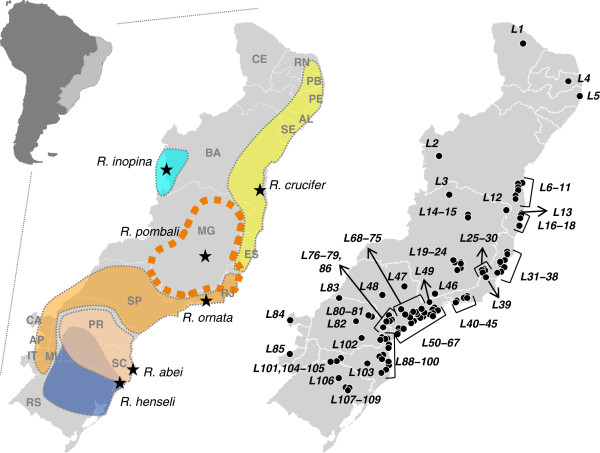
**Species distribution and sampling. **Distribution of morphological species in the *Rhinella crucifer* group, and localities sampled for this study: *Rhinella crucifer* in yellow, *R. inopina* in pale blue, *R. pombali* in dashed orange, *R. ornata* in solid orange, *R. abei* in pale orange, and *R. henseli* in dark blue. Stars indicate type localities. Initials indicate states or departments. Brazil: CE for Ceará, RN for Rio Grande do Norte, PB for Paraíba, PE for Pernambuco, AL for Alagoas, SE for Sergipe, BA for Bahia, MG for Minas Gerais, ES for Espírito Santo, RJ for Rio de Janeiro, SP for São Paulo, SC for Santa Catarina, and RS for Rio Grande do Sul. Paraguay: CA for Canindeyú, AP for Alto Paraguay, and IT for Itapuá. Argentina: MI for Missiones.

The *Rhinella crucifer* species group is a widespread group of toads that occurs throughout the Brazilian Atlantic Forest, a highly endangered biome [29] distributed along 30 lat degrees along the eastern coastline of South America. This widespread distribution includes high morphological variation, both within and among populations [30], which has led to a confused taxonomic history for the group [31]. In a taxonomic review, Baldissera et al. [30] recognized five species based on morphological and morphometric variation: *Rhinella crucifer* occurs from the State of Ceará to northern State of Espírito Santo; *R. pombali* is restricted to the State of Minas Gerais with isolated records in the state of Rio de Janeiro; *R. ornata* occurs from southern State of Espírito Santo to northern state of Paraná; *R. abei* is distributed from the State of Paraná to the State of Santa Catarina; and *R. henseli* occurs from southern State of Santa Catarina to the State of Rio Grande do Sul, with isolated records in the state of Paraná [30-33]. More recently, Vaz–Silva et al. [34] described the sixth species in the group, *Rhinella inopina*, with an allopatric distribution in forest enclaves within the Cerrado biome in the limits of the States of Goiás, Tocantins and Bahia [34] (Figure 1). A broad scale phylogeographical survey revealed that mitochondrial clades do not fully correspond to the current taxonomy [35]. *Rhinella henseli* corresponds to a highly divergent mitochondrial clade at the southern limit of the species’ range; a northern clade includes *R. crucifer* and part of *R. pombali*, and a central clade includes remaining populations of *R. pombali*, along with *R. ornata* and *R. abei*. *Rhinella pombali* is found along an east–west axis where the northern and central clades meet, ranging from the coast throughout the inland plateau. Its morphology is intermediate between *R. crucifer* and *R. ornata*[30], which taken together with the mitochondrial DNA is suggestive of a hybrid origin*.* Hybridization between the morphospecies *R. abei* and *R. henseli* is also possible as individuals occur in syntopy, and are thus likely to interbreed. Nuclear sequence data from this study showed only evidence of incomplete lineage sorting [35].

The lack of fine-scale sampling in Thomé et al. [35] prevented both detailed mapping of mtDNA lineages and description of geographic patterns of nuclear allele frequencies, resulting in an incomplete description of the spatial genetic structure in the *Rhinella crucifer* group. Here we greatly increase both the geographic and population sampling and use sequence data from a set of three mitochondrial and three nuclear genes to delimit genetic units in the *Rhinella crucifer* group. Given the widespread distribution of the group, and evidence of recent divergences and hybridization, we employ a combination of tree and allele frequency–based methods. We discuss the results in the context of available information on the taxonomy and history of the group, and provide a perspective on the potential for future evolutionary studies.

## Results

### Mitochondrial DNA genetic lineages

**Figure 2:**
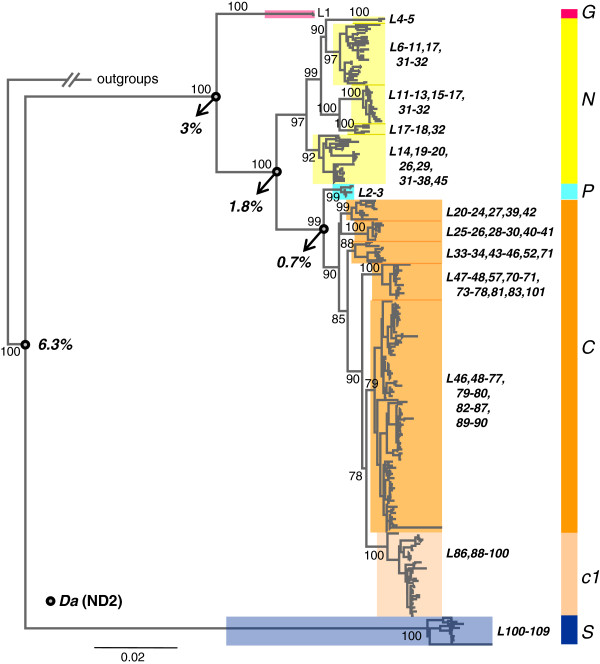
**Mitochondrial gene tree. **Mitochondrial haplotype tree inferred by maximum likelihood for the *Rhinella crucifer* group. Numbers before clades indicate support values, numbers after clades code for localities as in Figure 1. Values at nodes marked with circles indicate genetic distances (*Da*) between clades for the ND2 Fragment.

**Figure 3:**
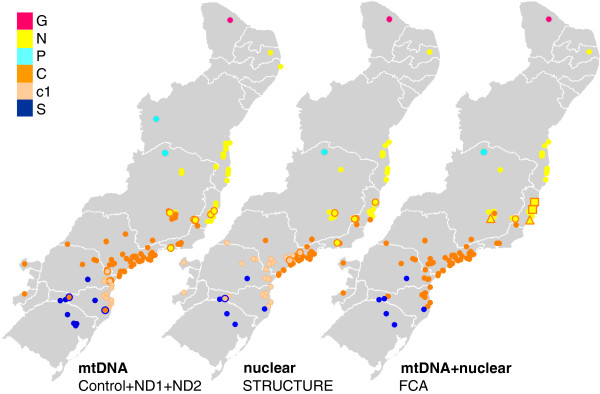
**Genetic units according to mitochondrial, nuclear and combined data. **Distribution of mitochondrial clades, nuclear demes, and FCA groups. Mitochondrial clade *G*, deme *G* and FCA group *G* in magenta; mitochondrial clade *N*, deme *N* and FCA group *N* in yellow; mitochondrial clade *P*, deme *P* and FCA group *P* in light blue; mitochondrial clade *C*, deme *C1* and FCA group *C* in orange; mitochondrial sub–clade *c1* and deme *C2* in pale orange; mitochondrial clade *S*, deme *S* and FCA group *S* in dark blue. Localities with more than one mitochondrial clade or more that one nuclear deme are coded with two colors. For the FCA map, squares, triangles and the two–coloured circle represent, respectively, localities where groups *C* and *N* co–occur, localities with individuals at the intersection between these two groups, and the locality where both conditions were observed.

We obtained 386, 401, and 398 sequences for control region, ND1, and ND2 fragments, respectively. After concatenation we found 305 unique haplotypes among the 404 sampled individuals. The topology of the mitochondrial tree (Figure 2) recovers the monophyly of the *Rhinella crucifer* group with high support. Two divergent clades within the group are located at the latitudinal extremes of the groups' distribution (Figure 3); the clade '*S*' is restricted to the southern region of the AF, states of Rio Grande do Sul, Santa Catarina and Paraná (L100−109), and the northernmost clade '*G*' is only found at the sampled locality of Guaramiranga, state of Ceará (L1). Haplotypes from the geographic region in between these two extremes form a main clade that contains most of the sampled haplotypes, ranging from the state of Santa Catarina to Paraíba (L2–101), which is itself structured with clades '*N*', '*P*', and '*C*'. Clade *N* has a northerly distribution (Figure 3) ranging from the state of Paraíba to the state of Rio de Janeiro, including the eastern and northeastern regions of the inland state of Minas Gerais (L4–20, 26, 29, 31–38, 45). This clade showed deep substructure with several well-supported subclades distributed sequentially from north to south, overlapping only at geographic transitions. Clade *P* is geographically restricted to two western localities in the states of Minas Gerais and Bahia (L2 and L3) (Figure 3). Clade *C* covers the central AF ranging from the state of Espírito Santo to the state of Santa Catarina, including most of the interior regions of the AF (western limits of the states of Minas Gerais and Bahia to Paraguay) (Figure 3). Substructure in this clade is evident, with several well-supported subclades including a derived subclade restricted to eastern localities of the states of Paraná and Santa Catarina (subclade *c1*). Other subclades showed partially or completely overlapping distributions mainly across the state of São Paulo and surroundings. Genetic distances (*Da*) based on the ND2 fragment ranged from 0.7% to 6.3% (Figure 2).

### Nuclear DNA

Among the 394 sampled individuals, we recovered 562 nuclear haplotypes for crystallin, 664 for rhodopsin, and 688 for alpha polypeptide, after phasing. The number of unique alleles for each gene was 25, 161, and 61, respectively. No recombination was detected by the DDS method or by permutation tests for the rhodopsin (D'=−0.15, p=0.08; G4=−0.12, p=0.11), crystallin (D='−0.02, p=0.17; G4=−0.02, p=0.19), or alpha polypeptide (D'=−0.03, p=0.14; G4=−0.05, p=0.08). The structure of allele trees shows poorly supported clades and polytomies across all genes (Additional file 1). Sharing of alleles among localities was frequent for the rhodopsin and alpha polypeptide, but less common for the crystallin marker (see Additional file 2 for allele lists).

**Figure 4:**
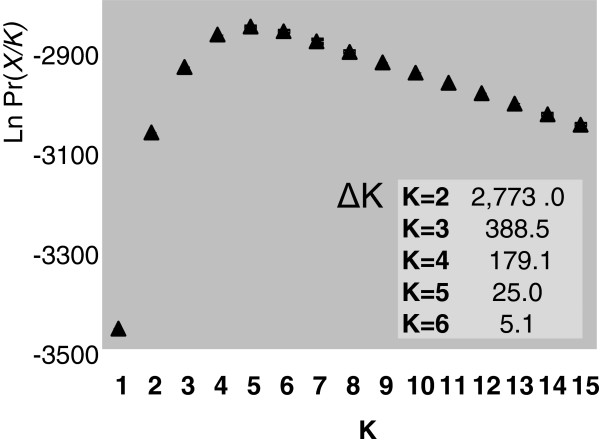
**K values curve. **Values of Ln Pr (X/K) for K=1–15 after analysis with STRUCTURE, and values of ΔK for K=1–6.

**Figure 5:**
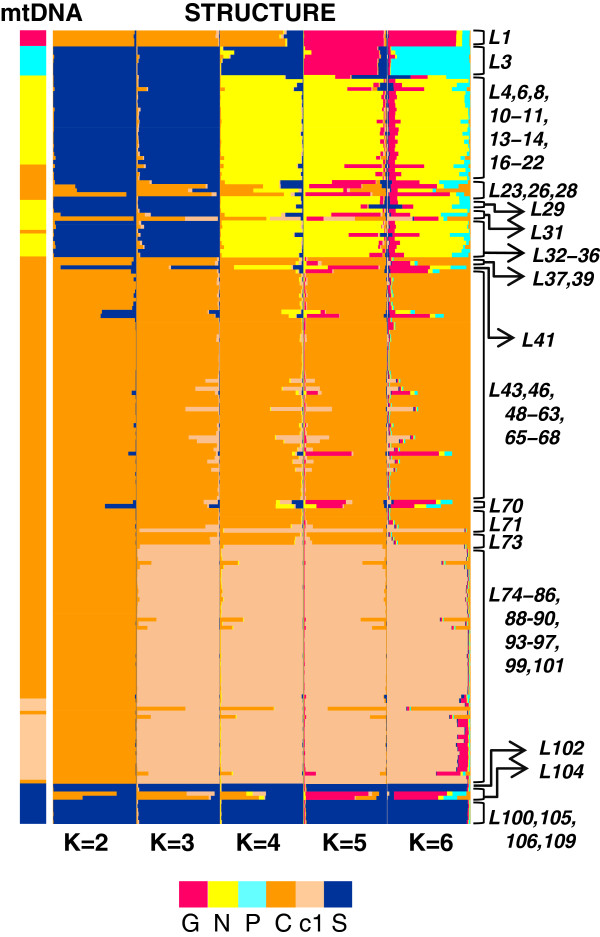
**Structure results. **STRUCTURE results for K=2–6. Individuals are represented as bars, with colors representing the proportion of assignment to each nuclear deme. Bars on the left indicate the respective mitochondrial main clades. Locality numbers follows Figure 1.

A complete dataset for the three nuclear fragments was available for 196 individuals from 83 localities. STRUCTURE results were evaluated according to the distribution of Ln Pr (*X*/*K*) and ΔK. Following these criteria, the values of K that best describe the structure of the nuclear data range from K=2 to K=6 (Figures 4 and 5). Five genetic breaks were consistently recovered indicating the clustering of individuals in six distinct geographical demes: deme '*G*' with individuals from the locality of Guaramiranga, state of Ceará (L1), deme '*P*' containing individuals restricted to western Bahia (L3), deme '*N*' clustering individuals from the state of Paraíba to the state of Rio de Janeiro including northern and eastern Minas Gerais (L4, 6, 8, 10–11, 13–14, 16–23, 26, 28–29, 31–39, 41), deme '*C1*' with individuals from the states of Espírito Santo, Rio de Janeiro, southern Minas Gerais, and the eastern region of the state of São Paulo (L23, 26, 28, 31, 37, 39, 43, 46, 48–63, 65–68, 70–71, 73), deme '*C2*' with individuals from the inland part of the state of São Paulo, states of Paraná and Santa Catarina (L71, 73, 74–86, 88–90, 93–97, 99, 101), and deme '*S*' with individuals from the states of Rio Grande do Sul, Santa Catarina and southern Paraná (L100, 102, 104–106, 109) (Figures 2 and 5). There are individuals from demes *N*, *C* and *S* with relatively high assignment values to deme *G*, which is explained by sharing alleles 1 and 2 of the rhodopsin locus (Additional file 2).

### Genetic units inferred from combined data

**Figure 6:**
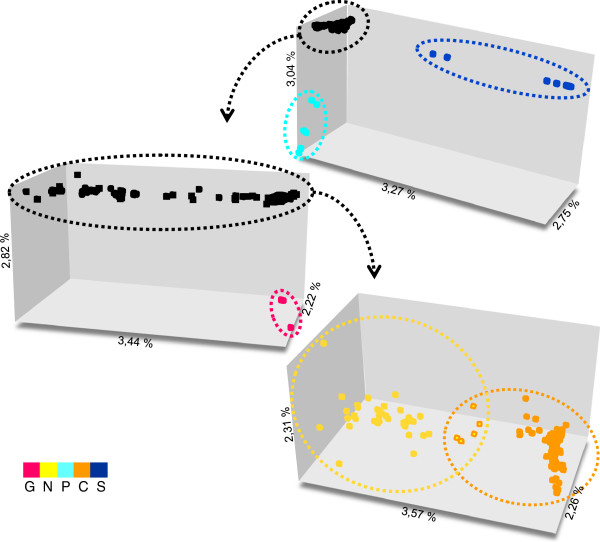
**FCA results. **FCA combining nuclear alleles and mitochondrial haplogroups. Group *S* in dark blue, group *P* in light blue, group *G* in magenta, group *N* in yellow, and group *C* in orange. Individuals in the intersection of groups *N* and *C* are represented by two-coloured squares.

The FCA performed with the complete nuclear dataset plus mitochondrial information showed individuals clustering in three groups corresponding to geographic regions: a group containing individuals from Rio Grande do Sul, Santa Catarina and Paraná states (group '*S'*, L100, 102, 104–106, 109), a group with individuals from western Bahia (group '*P*', L3), and a group containing the rest of the individuals. After the removal of groups *S* and *P*, the subsequent analysis shows the clustering of a third group with individuals from Guaramiranga (group '*G*', L1). In the analysis with the remaining individuals no more isolated groups were found. Instead, we found two partially overlapping groups, the first with individuals from the state of Paraíba to the state of Bahia (group '*N*', L4, 6, 8, 10–11, 13–14, 16–21, 28–29, 31–36) at one geographic extreme, and the second with individuals from the state of São Paulo to the state of Santa Catarina (group '*C*', L23, 26, 28, 39, 41, 43, 46, 48–63, 65–68, 70–71, 73–86, 88–90, 93–97, 99, 101) at the opposite geographic extreme of the population distribution. Individuals distributed in the intersection of both groups were from localities in the states of Espírito Santo and Minas Gerais (L22, 31, 33, 37) (Figure 6). Two individuals from group *S* show an intermediate position with group *C*+*N*, which is again explained by allele sharing at the rhodopsin locus (see above).

Taking together the results of the three analyses we delimited five genetic units: G (corresponding to the main clade *G*, deme *G* and group *G*), P (containing the subclade *P*, deme *P* and group *P*), N (with the main clade *N*, deme *N* and group *N*), S (corresponding to the main clade *S*, deme *S* and group *S*) and C (including the main clade *C*, demes *C1* and *C2*, and group *C*). Considering the analyses individually, uncertainty of limits occurred at boundary zones: i) we found overlaps between the mitochondrial clades *N* and *C* at L20, 26, 29, 33–34, 45, and *C* and *S* at L100 and 101; ii) localities with individuals assigned to more than one deme and individuals showing low coefficients of membership (q<0.85 considering K=2 to K=6) were more frequent at the boundary between demes *N* and *C1* (L20, 22–23, 26, 28, 31, 33, 37, 41, 43); individuals distributed at the intersection of FCA groups also occurred at this region (L22, 31, 33, 37). Discordance between results of distinct analyses also occurred at boundary zones. We found individuals with relatively high average membership coefficients (q≥0.85 considering K=2 to K=6) in the STRUCTURE analyses that pertained to conflicting mtDNA clades at localities L21–22 and L28–29.

## Discussion

Analyses of the genetic structure of mitochondrial and nuclear markers in the *Rhinella crucifer* species group revealed five distinct genetic units, three corresponding to the core geographic area of the group (N, C, and S), and two units with more isolated distributions (G and P). Overlap occurs between units N and C and between C and S, with evidence of some degree of admixture between those pairs. The concordance of genetic units with currently recognized species boundaries is very limited; the morphospecies with clear correspondence to the recovered genetic units are *R. henseli*, which is fully represented by unit S, and *R. inopina*, represented by unit P. Although we did find some correspondence between *R. crucifer* and unit N, and *R. ornata* and unit C, their distributions are not completely coincident. The distribution of *R. pombali* within the putative hybrid zone between N and C explains its intermediate morphology and corroborates that it does not have a distinct evolutionary history from neighboring units. *Rhinella abei* corresponds to a mtDNA sub-clade, but its correspondence to a genetic group in combined marker analyses is not well-supported and might depend on the inclusion of more markers. Finally, the morphological distinctiveness of populations in genetic units G remains to be tested.

### Delimitation of genetic units

We found deep mitochondrial structure within the *R. crucifer* group; haplotypes cluster in subclades that belong within the larger well-supported major clades *N, C, S, G* and *P*. Geographic distributions of sub–clades overlap greatly while major clades have more exclusive areas of occurrence. The mtDNA tree presented here (Figure 2) differs from the previously published topology [35]. All main clades (*N*, *C* and *S*) identified earlier were recovered by our current analysis, but the addition of previously unsampled geographically isolated populations revealed two new clades with apparently narrow distributions (clades *G* and *P*). Additional sampling also extended the known geographic distribution of clade *S* considerably to the north, the southern limit of clade *N* to the south, and the distribution of the *C* clade towards the southwest (Figure 3). Our analyses also improved the support for relationships within clade *C*, especially for northern sub–clades that showed more restricted distributions than sub–clades in the state of São Paulo and western regions of the states of Paraná and Santa Catarina, which overlapped extensively. However, this pattern might reflect unbalanced sampling. In eastern regions of the states of Paraná and Santa Catarina individuals clustered within sub–clade *c1*, a pattern that was recovered before [35]. The overlapping geographic distributions of all *N* sub–clades confirmed that their allopatric distributions inferred in Thomé et al. [35] was an artifact of less-extensive sampling.

The nuclear gene genealogies (Additional file 1) confirmed incomplete lineage sorting previously described for the *Rhinella crucifer* group [35]. The nuclear genetic structure was assessed from Bayesian individual assignment using the method of Pritchard et al. [22] with three nuclear loci and a model without prior information on the origin of individuals. This model avoids circularity in cross–validating results with other evidence (i.e. geography and mtDNA structure) but the limited number of loci can potentially reduce the power of reliably inferring the optimal number of clusters. We therefore delimited an interval of possible values of K (K=2–6) by comparing scores produced by different criteria [ΔK, Ln Pr(*X/K*)]. Assignment results consistently recovered five genetic breaks and six demes could be delimited based on the nuclear data alone (Figure 5). Individuals with low average assignment coefficients were primarily restricted to the geographic boundaries of demes (Figures 1 and 5), which argues for admixture at contact zones between divergent lineages. An important exception are the two individuals from L104 that were assigned, albeit not strongly, to deme *G*. This is certainly not due to admixture resulting from secondary contact between diverged lineages because demes *G* and *S* do not share a contact zone. This result may be rather explained by shared ancestral polymorphism, especially at the rhodopsin locus (the case of widespread alleles 1 and 2; see Additional file 2).

Clusters defined by assignment analyses of nuclear markers correspond well to the mtDNA topology; of the six inferred demes, *G*, *N*, *P*, and *S* correspond largely to main mitochondrial clades *G*, *N*, *P*, and *S*, and demes *C1* and *C2* together account for the mitochondrial clade *C* (Figure 3). Substructure in the mitochondrial clade *C* does not correspond to the genetic break between demes *C1* and *C2*. However, at values of K ≥7 a cluster geographically coincident with the distribution of mtDNA sub–clade *c1* was revealed (data not shown), which suggests that additional data (more nuclear markers) will likely uncover a nuclear genetic structure consistent with that of mtDNA for the eastern regions of the states of Paraná and Santa Catarina.

The limited number of loci used in the STRUCTURE analysis limits the power of our results; clearly more conclusive inferences will require additional markers. Nonetheless, we were surprised at the performance of this method with only three loci, suggesting that they contain the signature of geographic history of the major lineages. Other studies with similar number of markers coded from sequence data have found similar results: assignment analyses based on five nuclear loci successfully revealed cryptic species in the West African forest gecko *Hemidactylus fasciatus*[36]. Success in detecting relevant genetic structure with a reduced number of loci in assignment analysis (up to two orders of magnitude less that in regular analysis) may depend on marker choice, sufficient sampling of individuals, as well as the specific history of the focal species; optimal clustering may be achievable as long as the choice of markers is efficient in targeting the question to be addressed [28]. In the present case, the use of more conserved markers may have prevented revealing further genetic structure (e.g., at the population level) that would be predictable for an amphibian [37]. Finer scale genetic structure, albeit interesting in itself, could have resulted in oversplitting the species group into more genetic units with no association with lineage/species diversification. In this context, a comparative study of the sensitivity of genetic assignment analyses to marker choice should be of great utility in studies concerning the boundaries of closely related species.

The combination of mitochondrial and nuclear data in FCA corroborated the results obtained for these two marker classes when analyzed individually (Figure 6). Therefore, the existence of five genetically-defined units within the *Rhinella crucifer* group is strongly supported by the overall genetic data. The units C, N and S are parapatrically distributed spanning the main range of the group. Units G and P have very restricted distributions at the northern and western limits of the range, and appear to be geographically isolated. These allopatric distributions likely reflect the patchy nature of the habitat in the transition zones between the Atlantic Forest and neighboring biomes (Caatinga to the north and Cerrado to the west), but broader-scale sampling might also explain this isolation pattern to some degree.

### Two potential hybrid zones

Geographic overlap of units occurs along the borders of units N and C and more extensively between units C and S (Figure 3). Strong evidence of admixture was found for the N/C contact zone, with four individuals showing hybrid cyto–nuclear allele combinations. These individuals were distributed in localities along an east–west axis near the limits of the states of Espírito Santo, Rio de Janeiro, and Minas Gerais. Although these four samples are spatially restricted relative to the ranges of units C and N, the hybrid zone between those units may be wider across the transition zone. This is supported at the level of both mtDNA and nuclear data given the distribution of admixed individuals in areas of clade overlap (Figure 3) and by the lack of clear delimitation in the FCA groups in combined analysis (Figures 3 and 6). We therefore predict that the hybrid zone between units N and C extends across most of the states of Espírito Santo and southern Rio de Janeiro, in addition to the central region of the state of Minas Gerais.

Evidence for a second hybrid zone between units C and S is less conclusive. The co–occurrence of individuals belonging to mitochondrial clades *C* and *S* was detected at two localities (L100 and 101, Figure 1), with individuals of both clades reproducing synchronously at the same pond at locality L100 (L. M. Giassom, personal communication). We sampled seven individuals at this pond but obtained a complete nuclear dataset for only one individual, showing no signal of hybridization both with STRUCTURE and FCA. The remaining six individuals with incomplete nuclear datasets (two of the three nuclear fragments sequenced) were not included in STRUCTURE and FCA analyses but their allelic compositions provided some evidence of mixing at this locality (Additional file 2). Sharing of nuclear alleles occurred exclusively within members of the same mitochondrial clade with the exception of one individual from clade *C* that contained alleles otherwise exclusively found in members of clade *S* (Additional file 2). This signal of admixture was evident at the two nuclear markers available for that individual (rhodopsin and alpha polypeptide), suggesting that incomplete sorting of lineages is, in this case, a much less parsimonious explanation than recent hybridization. Contrastingly, the two individuals from locality L104 (Figure 1) with low assignment to deme *S* (moderately assigned to deme *G*) cannot be taken as evidence of admixture, as discussed above.

Admixture between the closely related genetic units in the *Rhinella crucifer* group is not surprising; natural hybridization in bufonids is very frequent and cases of natural hybrids have been reported in genetic studies for almost every continent in which this cosmopolitan family occurs [38-41]. In our case, we found stronger evidence for hybridization between units N and C than between units C and S, a result that might be explained by the divergences among these clades. Malone & Fontenot [42] revisited a large dataset of experiments crossing species from all major clades of bufonids [43] and re–interpreted results in the light of newly available phylogenies. The authors found intrinsic postzygotic reproductive isolation to be a gradual and likely outcome related to the degree of divergence, with high levels of divergence (~9% 12S and 16S) being required for hybrid offspring mortality during early developmental stages. Because the levels of divergence found in the *R. crucifer* complex are lower than those found by Malone & Fontenot [42], and considering that the 12S and 16S have lower mutation rates than the mitochondrial markers used in this study (0.0025 substitutions per site per million years for 12S and 16S, compared to 0.0096 for the ND2 fragment [44,45], the relevant question is if the levels of mtDNA divergence observed between the S unit and all other units (6.3% ND2, Figure 2) are sufficient to produce genetic isolation. If so, the expectation that fitness should be lower for crosses between C and S than N and C could be tested in a future study comparing levels of hybridization at the respective hybrid zones. Colliard et al. [46] found slight fitness reduction in F1 hybrids, strong hybrid breakdown in backcrossed offspring, and complete mortality in F2 hybrids for Plio–Pleistocene diverged populations of toads from the *Pseudepidalea viridis* group. However, hybridization was a weak indicator of phylogenetic relationship for the African 20-chromosome toads, and seems to be widespread among all species in that group independent of phylogenetic distance [40].

### A problematic taxonomy

Six species of the *Rhinella crucifer* group are currently recognized on the basis of morphological and morphometric data [30,31,34], although diagnosis of these morphospecies is not always straightforward. The results of this study underscore the fact that current classification does not reflect evolutionary relationships within the group [35], and further identifies several groups or populations that are especially problematic. Unequivocal correspondence between genetically cohesive units and recognized species exists only between unit P and *R. inopina*, and between S and *R. henseli*. *Rhinella henseli* corresponds to a deeply divergent mitochondrial lineage [35] and is the most clearly diagnosable species in this complex [30]. Strong but problematic associations are evident between unit N and *R. crucifer*, and between unit C and *R. ornata*. These species are easily diagnosable morphologically if one compares individuals from across the core ranges of units C and N (i.e., not including individuals from the putative hybrid zone). The difficulty is that other named species appear to be nested within these 2 units. This is the case for *R. abei*, a species that occurs within the distribution of unit C, and which shows very subtle diagnostic characters [30]. The morphospecies *Rhinella abei* does not correspond to a genetic unit but its distribution roughly coincides to subclade *c1*. Interestingly, Baldissera et al. [30] found a similar pattern in morphometric analysis in that the distribution of *R. abei* occurs within the polygon of the wider distribution of *R. ornata*. Despite the morphological data, we cannot decidedly comment on the status of *R. abei*, because the limited number of nuclear markers in our analysis may contribute to our inability to detect this species (see above). A second difficulty arises in the correspondence between unit C and *R. ornata*. The type locality for this species ("probably Rio de Janeiro" according to Bokermann [47]) and type localities for all its synonyms [31] lies within the putative hybrid zone between unit N and C; thus, the type series may actually include hybrids. A careful examination of the type series is necessary to solve this issue.

Hybridization is probably the cause of the most problematic case in the taxonomy of the *R. crucifer* species group. *Rhinella pombali* was originally described based on individuals from the central region of the state of Minas Gerais [30]. This species’ distribution has since been expanded to the state of Rio de Janeiro near the border with Espírito Santo [33], resulting in an overall area of occurrence that is largely concordant with the putative hybrid zone between units N and C. The morphology of *R. pombali* shows characters that are intermediate between *R. crucifer* and *R. ornata* (tarsal tubercles forming a row in *R. ornata* and in small individuals of *R. pombali*; tarsal tubercles forming a fringe in *R. crucifer* and in large individuals of *R. pombali*) [30]. This species has an intermediate size as well, which can mislead the interpretations of traditional morphometric analysis (Figure 27 in Baldissera et al. [30]). Projected principal component scores of *R. pombali* occupied a distinct area of the morphometric space but most of the variation contained in the canonical axis is explained by body size and not by significant changes in shape. Not surprisingly, the advertisement call of allopatric populations of *R. pombali* and *R. ornata* overlap in call duration and dominant frequency [48], but there is no available data on the advertisement call of *R. crucifer*. Our data argue in favor of the hypothesis that *R. pombali* was described based on hybrids between *R. crucifer* and *R. ornata*[35]. Similar difficulties in inferring species within closely related Bufonidae have been previously attributed to hybridization by taxonomists and phylogeneticists [38,49] with toads being referred as a test–case in anuran systematics [40]. The International Code for Zoological Nomenclature [50] states that names proposed for hybrid specimens are excluded from the provisions of the Code (article 1.3.3) and must not be used as the valid name for either of the parental species, regardless of precedence, achieving a condition of homonymy (article 23.8). Therefore, the name *R. pombali* (and combinations) should be considered a synonym of both *R. crucifer* and *R. ornata*. Similarly, the name proposed for hybrids resulting from crosses between *Pelophylax lessonae* and *P. ridibundus* ("*Rana esculenta*"), remains under synonymy of the names of both parental species [31,51], although in this case the molecular evidence confirming the hybrid condition came latter [52].

Further investigation of the status and distribution of genetic unit G will be another requirement to reach a taxonomical consensus for this group. If careful inspection of specimens from this region reveals considerable morphological divergence, then this population may justify the description of a new species under morphological and phylogenetic criteria.

### Implication for evolutionary studies and conservation

Genetic diversification in the *Rhinella crucifer* group dates back to the Plio-Pleistocene and earlier studies based on the geographic distribution of mtDNA lineages suggested a major role of geographic barriers as promoters of divergence [35]. However, that first broad survey of genetic diversity included limited and uneven sampling, and thus necessarily precluded inferences on the demographic history of the group. Recent studies of Atlantic Forest taxa support a scenario of regional differences in evolutionary forces promoting diversification in this biome [53,54] resulting in patterns that are hard to interpret or generalize for the whole biome (but see Carnaval et al. [55]). Both fine scale sampling and the delimitation of units in this study will enable inferences of demographic patterns at regional scales, contributing to a better understanding and hypothesis testing of the microevolutionary processes affecting the *R. crucifer* group.

The genetic delimitation of units within the *R. crucifer* also revealed two hybrid zones that may have originated through differentiation under gene flow or from secondary contact after a long period of isolation. While recent studies suggest a history of past habitat fluctuations for the Brazilian Atlantic Forest biome throughout the Pleistocene to the Holocene [35,55], it is reasonable to consider a role of more recent human-induced habitat alterations in bringing toads into sympatry [56], even though the *Rhinella crucifer* group occurs exclusively in forested habitat. In a landscape ecology study, Dixo et al. [57] noted that *R. ornata* occurs at higher densities in medium sized fragments than in continuous forest or small fragments, raising the possibility that populations increase and expand distributions under moderate disturbance. Hybridization in Bufonids has been useful for studying mechanisms of reproductive isolation and speciation [42,43,46]. In Neotropical toads. it was shown by Haddad et al. [58] and suggested by karyotype data [59], but as of yet, we still do not have a good description of hybrid zone dynamics in the Neotropics. The two putative hybrid zones we identified enable experiments and hypothesis testing on hybrid fitness relative to genetic divergences, population history, and on the role of anthropogenic disturbances. Thus, the *Rhinella crucifer* species complex represents an excellent candidate system for studies of how hybridization contributes to diversity and to biological complexity in the Atlantic Forest biome.

The six species currently recognized in the *Rhinella crucifer* group are endemic to one of the most biodiverse and endangered global hotspots [60]. None of the species are considered threatened or endangered according to the IUCN red list [61], but our results indicate that there are still cryptic lineages and evolutionary processes to be described and preserved in this group. The most recent survey of remaining areas of the Brazilian Atlantic Forest points to a loss of at least 85% relative to the original distribution, and raises concerns about the inefficiency of the current system of conservation units in keeping fragments of considerable size and connectivity [29]. The maintenance of genetic diversity, and especially of continued evolutionary potential, has been typically neglected in conservation policies in this biome [55]. Our study contributes the identification of a previously unidentified new lineage with a restricted distribution.

## Conclusions

The existence of five genetic units within the *Rhinella crucifer* species group is supported by mitochondrial and nuclear data, with three units composing the core geographic distribution of the group and two units located at extremes of the range. Evidence for hybridization was found between two pairs of units although cyto–nuclear mixing was restricted to only the less divergent pair. The concordance between inferred genetic units and previously recognized species was limited and sets more accurate distribution limits for *R. crucifer* and *R. ornata.* Our data indicate that *R. pombali* is not a valid species, and underscore the need for examining hybridization in the type series of *R. ornata.* The recognition of *R. abei* as an independent unit will rely on more markers, while the morphological distinctiveness of genetic unit G indicates that more genetic units may still await formal taxonomic recognition. Our results clarify the genetic structure within the *R. crucifer* complex and set the framework for further work on the mechanisms of evolutionary diversification, hybridization and biological conservation in the endangered Atlantic Forest.

## Methods

### Sampling and molecular protocols

We pooled sequences from 339 newly collected individuals (GenBank Accession numbers KC198085–KC199966) with previously published sequences from 65 individuals (GenBank Accession numbers GU907122–GU907480) [35] for a total of 404 individuals from 109 localities (Additional file 2), encompassing most of the distribution of this species group (Figure 1). We included fragments from 3 previously characterized mitochondrial and 2 nuclear genes [35] and one additional nuclear fragment. The sequenced mitochondrial fragments included i) the control region and a short segment of the adjacent cytochrome b gene (referred to as the control region, 935 bp), ii) a fragment including 50 base pairs of the 16S gene, the complete tRNALeu, the complete NADH dehydrogenase subunit 1, the tRNAIle and part of the tRNAGln genes (referred to as the ND1, 1350 bp), and iii) a partial sequence of the NADH dehydrogenase subunit 2 (referred to as the ND2, 906 bp). The nuclear fragments were segments of i) exons 1 and 4 of the rhodopsin gene (referred to as the rhodopsin, 279 bp), ii) the β-crystallin gene (referred to as the crystallin, 357 bp), and iii) the intron 1 of the A alpha polypeptide (referred to as the alpha polypeptide, 707 bp, [62]).

We digested tissue samples and extracted whole genomic DNA using QIA Quick DNEasy kits following the manufacturer’s protocol (Qiagen Inc.). We amplified target fragments with polymerase chain reactions (PCR) using one microliter of the eluted extract (~ 1–10 ng DNA) as template. Amplification conditions included an initial denaturation step at 94°C (5 min) followed by 35 cycles consisting of denaturation at 94°C (1 min), annealing at 48.3–60.2°C (1 min), extension at 72°C (1 min), and a final extension step at 75°C (5 min). Amplicons were purified with 10 units of Exonuclease I (Exo I) and one unit of shrimp alkaline phosphatase (SAP) as template for sequencing reactions. We used the same amplification primers for sequencing using Big Dye termination sequencing chemistry (Applied Biosystems). We purified sequencing products using Sephadex G-50 columns and electrophoresed on ABI PRISM 3100 or 3730 Genetic Analyzers (Applied Biosystems). We checked electropherograms for errors, heterozygotic positions, and indels in the assembled contiguous sequences. A subset of the nuclear fragments (108 individuals for the crystallin and 11 for the alpha polypeptide) containing either multiple heterozygotic positions or insertions/deletions were cloned using the pGEM-T Vector System (Promega Corporation) and transformed into One Shot TOPO10 competent *Escherichia coli* (Invitrogen Corporation). After cloning we amplified the desired fragments directly from transformed colonies and sequenced as described above. Potential cloning errors were eliminated by comparing the heterozygotic sites of cloned sequences with those of the original sequences. We aligned all contigs with ClustalW [63] and checked alignments by eye.

### Delimitation of genetic units

We used three methods to assess genetic structure and identify clusters of individuals corresponding to evolutionary units: we i) constructed phylogenetic trees, ii) performed population assignment analyses based on allele frequencies, and iii) checked for correspondence between genotypes. Final evaluation of species limits was achieved by comparing the clusters detected by the different methods.

For the mitochondrial data we constructed a haplotype tree by concatenating the three fragments and including *Anaxyrus americanus*, *Rhinella icterica, R. rubescens, R. granulosa, R. schneideri* and *R. marina* as outgroups, based on molecular and morphological data [64-66]. We used maximum likelihood in the program RAxML [67] with the GTR model and CAT approximation of rate heterogeneity [68] and ran 10 replicates 1,000 bootstrap repetitions to infer nodal support. We divided the alignment in 8 partitions as follows: control region, first, second and third codon positions of the coding region of the ND1 fragment, remaining regions of the ND1 fragment, and the first, second and third codon positions of the ND2. We also estimated net sequence divergences (*Da*) [69] between major clades in DNAsp 5.10.01 [70].

For the nuclear dataset we first resolved heterozygotic positions using the coalescent-based Bayesian method PHASE 2.1 [71] implemented in DNAsp 5.10.01 [70], setting the number of interactions to 1,000, burnin to 1,000, and thinning interval to 01. We manually excluded from further analyses the sequences phased with a posterior probability inferior to 0.9. We then tested the three markers for intragenic recombination by checking the correlation between linkage disequilibrium (LD) and physical distance using two permutation tests: D−prime (D', [72]) and the four−gamete test (G4, [73]). Both tests were performed in the OmegaMap 0.5 program [74] using 100,000 permutations. We also used the Difference of Sums of Squares (DSS) test implemented in TOPALi v. 2.5 [75]. This program slides two windows along the alignment (a left−hand and right−hand window) and calculates the sum−of−squares (SS) between observed genetic distances and distances based on an estimated tree. By comparing the SS of the left window to the right window the program infers putative recombination breakpoints. We included all sequences using a window size of 93 bp, steps ten bp long, and 100 bootstrap repetitions.

We used allele trees and population assignment analyses to assess genetic structure in the nuclear sequence data. We constructed trees for each nuclear marker using maximum likelihood with the same parameters applied to the mtDNA data. For population assignment analysis we used the algorithm implemented in the program STRUCTURE 2.3 [21] coding phased sequences as alleles, including only individuals with a complete nuclear dataset (3 nuclear fragments). We used the admixture model with independent allele frequencies keeping lambda = 0.7 after preliminary tests. We discarded 1,000,000 iterations as burnin and counted the next 3,000,000 iterations as our run. We considered 1 to 15 as a plausible range of putative populations (K) and performed 20 replicates for each K value, using CLUMPP 1.1.2 [76] to find the optimal match of runs with the Greedy and LargeKGreedy algorithms and 1,000,000-200,000 random input orders tested. Because of the small number of nuclear loci, we avoided making inferences on optimal clustering. Instead, we observed the genetic breaks that appeared repeatedly over suitable values of K. To identify the most suitable values of K we plotted the average log likelihood values [Ln Pr (*X*/*K*)] for each K, calculated ΔK values [77] with Structure Harvester v0.6. [78] and checked for biologically meaningful population clusters.

We combined phased nuclear sequences and the mitochondrial data (a total of 4 loci) in a three-dimensional factorial correspondence analyses (FCA, [79]) using GENETIX 4.05 [80]. FCA uses correspondence among genotypes to graphically plot individuals in a three-dimensional hyperspace based on their allele frequencies, thus permitting combined analysis of all markers to identify genetic structure with no *a priori* information. Because of low levels of haplotype sharing in the mitochondrial data, we coded well-supported major clades as alleles (clades '*S*', '*G*', '*N*', '*P*', and '*C*', see results). We performed hierarchal FCA runs, starting with the complete dataset and removing individuals forming divergent groups systematically until groups were no longer separable.

## Competing interests

The authors have no financial or other competing interest to declare.

## Authors’ contributions

MTCT, KRZ, CFBH and JA designed the study and collected the samples in the field. MTCT produced the molecular data, performed the analysis and drafted the paper. JA, KZ and CFBH revised the manuscript. All authors approved the final manuscript.

## Supplementary Material

Additional file 1 **Nuclear gene genealogies. **Numbers before clades indicate support values (values under 30 are not shown). Click here for file

Additional file 2 **Individuals samples in this study. **Columns indicate voucher or tissue number, localities (with codes), sequenced mtDNA fragments, haplotypes, mitochondrial clade, nuclear alleles and individual codes for the STRUCTURE analysis. Click here for file
