# Supplementary material for: Delimiting genetic units in Neotropical toads under incomplete lineage sorting and hybridization
Source: BMC Evol Biol. 2012 Dec 11;12:242. doi: 10.1186/1471-2148-12-242 (PMC3574056; doi:10.1186/1471-2148-12-242)
Supplement: Additional file 1 — Nuclear gene genealogies. Numbers before clades indicate support values (values under 30 are not shown). [file 1471-2148-12-242-S1.pdf]

Additional file1: Maximum likelihood trees for nuclear fragments. Numbers before clades indicate support values (bootstrap). Values under 30 are not shown.

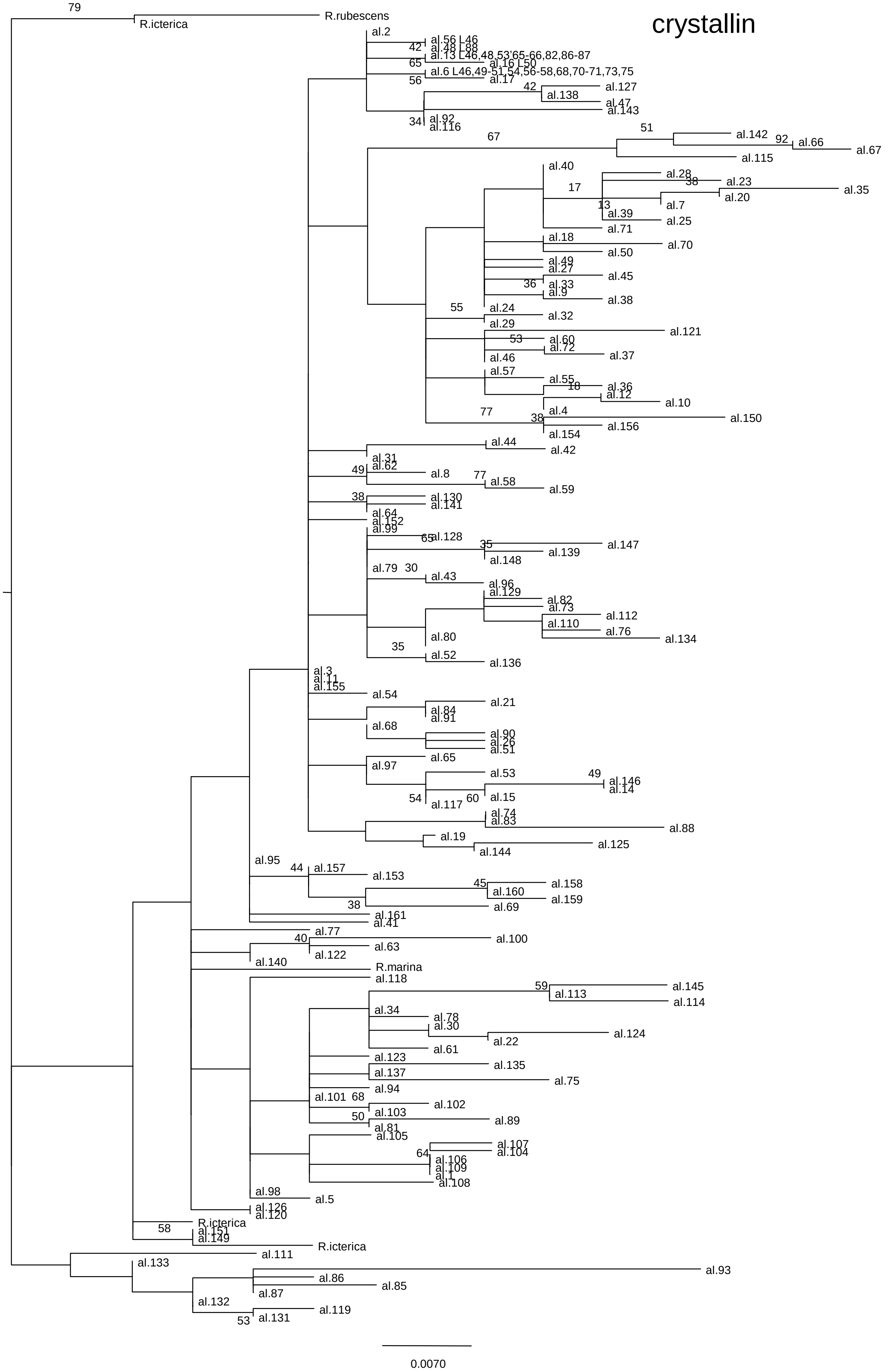

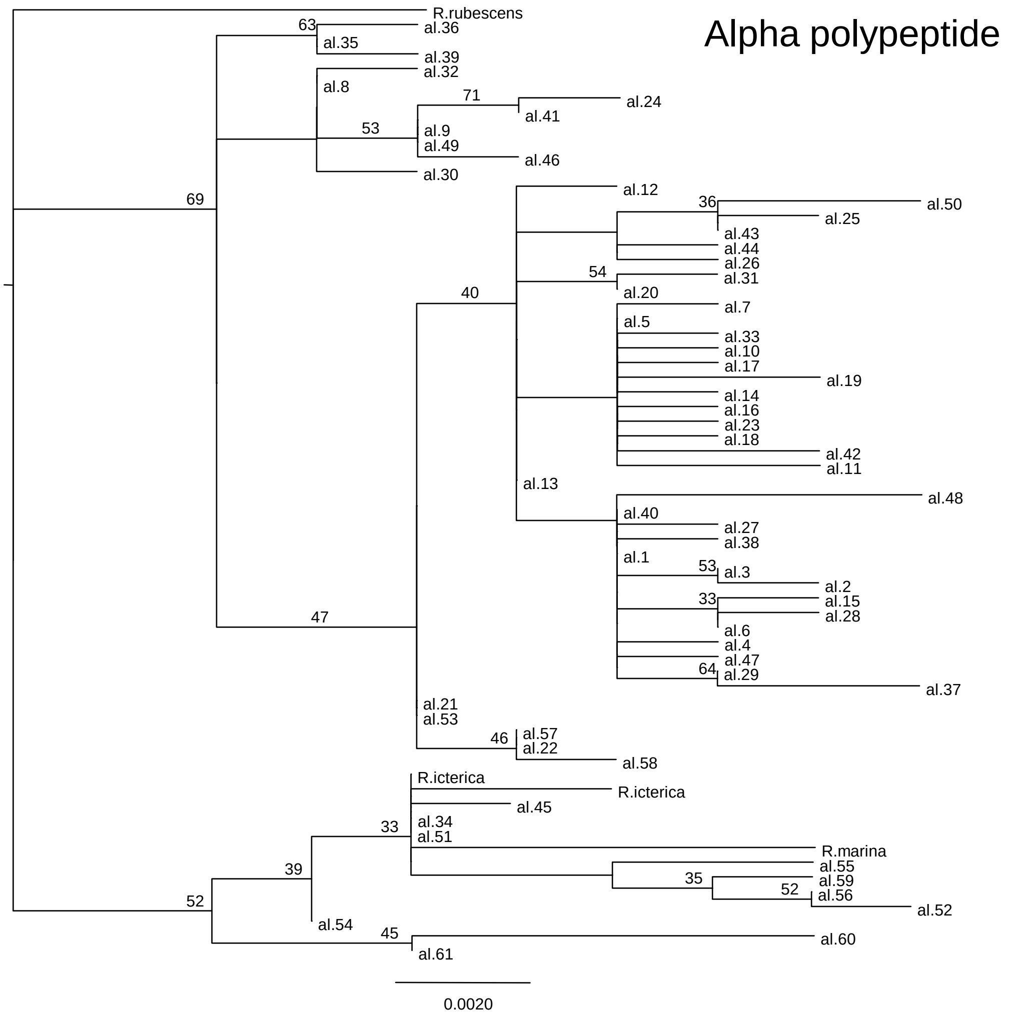
Alpha polypeptide
R.rubescens
63
al.36
al.35
al.39
al.32
al.8
71
al.24
al.41
53
al.9
al.49
al.46
al.30
69
al.12
36
al.50
al.25
al.43
al.44
al.26
54
al.31
al.20
40
al.7
al.5
al.33
al.10
al.17
al.19
al.14
al.16
al.23
al.18
al.42
al.11
al.13
al.48
al.40
al.27
al.38
al.1
53
al.3
al.2
33
al.15
al.28
al.6
al.4
al.47
64
al.29
al.37
47
al.21
al.53
46
al.57
al.22
al.58
R.icterica
R.icterica
al.45
33
al.34
al.51
R.marina
al.55
35
al.59
52
al.56
al.52
39
52
al.54
45
al.60
al.61
0.0020

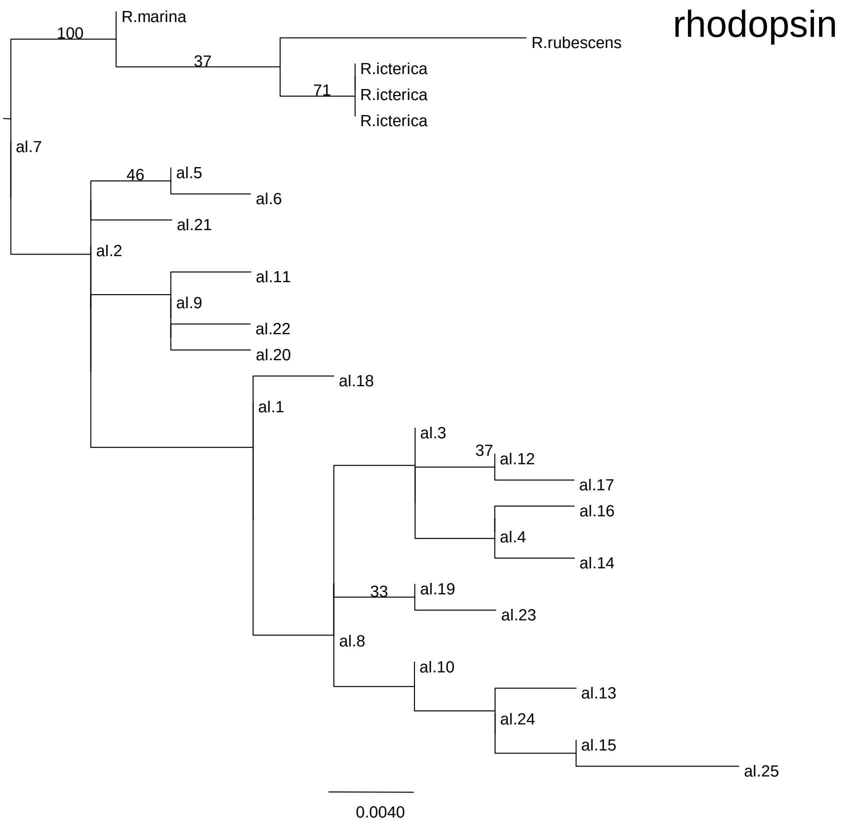
rhodopsin
R.marina
100
37
R.rubescens
R.icterica
71
R.icterica
R.icterica
al.7
46
al.5
al.6
al.21
al.2
al.11
al.9
al.22
al.20
al.18
al.1
al.3
37
al.12
al.17
al.16
al.4
al.14
33
al.19
al.23
al.8
al.10
al.13
al.24
al.15
al.25
0.0040
