# Supplementary material for: Delimiting genetic units in Neotropical toads under incomplete lineage sorting and hybridization
Source: BMC Evol Biol. 2012 Dec 11;12:242. doi: 10.1186/1471-2148-12-242 (PMC3574056; doi:10.1186/1471-2148-12-242)
Supplement: Additional file 2 — Individuals samples in this study. Columns indicate voucher or tissue number, localities (with codes), sequenced mtDNA fragments, haplotypes, mitochondrial clade, nuclear alleles and individual codes for the STRUCTURE analysis. [file 1471-2148-12-242-S2.pdf]

Additional file 2: Individuals samples in this study. Columns indicate voucher or tissue number, municipality and state of the sampled locality, locality code (L), sequenced mtDNA fragments, mtDNA haplotype (Hap.), mtDNA main clade or subclade (clade), allele number for alleles 1 and 2 of sequenced nuclear fragments, and individual number in the STRUCTURE analysis (STR).

				**mtDNA**					**nuclear**				
							**a.polypep.**		**crystallin**		**rhodopsin**		**STR**
**number**	**municipality**	**state**	**L**	**fragments**	**Hap.**	**clade**	**al.1**	**al.2**	**al.1**	**al.2**	**al.1**	**al.2**	
CFBH-T10831	Guaramiranga	CE	1	control,ND1,ND2	hap1	G	1	2	1	1	1	2	1
CFBH-T10832	Guaramiranga	CE	1	control,ND1,ND2	hap1	G	3	3	1	1	1	2	2
CFBH-T10833	Guaramiranga	CE	1	control,ND1,ND2	hap2	G	1	2	1	1	2	2	3
CFBH-T10834	Guaramiranga	CE	1	control,ND1,ND2	hap1	G	1	2	1	1	2	2	4
CFBH-T10841	Guaramiranga	CE	1	control,ND1,ND2	hap1	G	-	-	-	-	-	-	-
PHV1450	São Desidério	BA	2	ND1,ND2	hap289	P	-	-	150	150	20	20	-
PHV1451	São Desidério	BA	2	ND1,ND2	hap290	P	-	-	150	150	2	24	-
MTJ26	Januária	MG	3	control,ND1,ND2	hap285	P	48	49	150	150	20	11	5
MTJ27	Januária	MG	3	control,ND1,ND2	hap286	P	-	-	150	150	2	24	-
MTJ53	Januária	MG	3	control,ND1,ND2	hap285	P	9	9	150	150	20	11	6
MTJ54	Januária	MG	3	control,ND1,ND2	hap287	P	1	9	150	150	2	24	7
MTJ55	Januária	MG	3	control,ND1,ND2	hap286	P	-	-	150	150	2	24	-
MTJ56	Januária	MG	3	control,ND1,ND2	hap285	P	50	48	-	-	-	-	-
MTJ71	Januária	MG	3	control,ND1,ND2	hap285	P	48	48	150	150	2	24	8
MTJ72	Januária	MG	3	control,ND1,ND2	hap288	P	48	48	150	150	7	7	9
MTJ84	Januária	MG	3	control,ND1,ND2	hap287	P	48	49	150	150	20	20	10
MTJ85	Januária	MG	3	control,ND1,ND2	hap285	P	48	48	150	150	20	20	11
CFBH-T3642	Areia	PB	4	control,ND1,ND2	hap222	N	37	36	106	107	8	8	12
CFBH-T3645	Recife	PE	5	control,ND1,ND2	hap223	N	-	-	108	109	11	11	-
CFBH-T3650	Recife	PE	5	control,ND1,ND2	hap223	N	-	-	110	111	2	2	-
CFBH-T3658	Recife	PE	5	control,ND1,ND2	hap223	N	-	-	1	1	2	2	-
CFBH-T3663	Recife	PE	5	control,ND1,ND2	hap223	N	-	-	112	113	2	2	-
CFBH-T9161	Itacaré	BA	6	control,ND1,ND2	hap248	N	5	37	129	129	11	11	13
MNRJ35341	Itacaré	BA	6	control,ND1,ND2	hap273	N	-	-	141	142	11	11	-
MNRJ35342	Itacaré	BA	6	ND1,ND2	hap274	N	37	35	-	-	11	11	-
CFBH-T9233	Aurelino Leal	BA	7	control,ND1,ND2	hap280	N	-	-	117	146	11	11	-
CFBH-T4151	Uruçuca	BA	8	control,ND1,ND2	hap232	N	35	35	118	119	11	21	14
CFBH-T9129	Uruçuca	BA	8	control,ND1,ND2	hap244	N	5	36	-	-	2	2	-
CFBH-T9131	Uruçuca	BA	8	ND1,ND2	hap245	N	42	35	126	105	-	-	-
CFBH-T9132	Uruçuca	BA	8	control,ND1,ND2	hap246	N	37	39	128	5	2	22	15
CFBH-T9140	Uruçuca	BA	8	control,ND1,ND2	hap247	N	35	36	-	-	2	11	-
CFBH-T9141	Uruçuca	BA	8	control,ND1,ND2	hap248	N	42	37	-	-	11	11	-
CFBH-T9147	Uruçuca	BA	8	control,ND1,ND2	hap249	N	5	36	-	-	1	22	-
CFBH-T9176	Uruçuca	BA	8	ND1,ND2	hap245	N	1	35	-	-	11	11	-
CFBH-T9181	Uruçuca	BA	8	control,ND1,ND2	hap249	N	36	39	-	-	11	11	-
CFBH-T3572	Itabuna	BA	9	ND1,ND2	hap214	N	35	36	-	-	11	11	-
CFBH-T3577	Itabuna	BA	9	control,ND1,ND2	hap215	N	-	-	-	-	11	11	-
CFBH-T3578	Itabuna	BA	9	control,ND1,ND2	hap216	N	36	36	-	-	11	11	-
CFBH-T3586	Itabuna	BA	9	ND1	hap217	N							-
CFBH-T3591	Itabuna	BA	9	control,ND1,ND2	hap218	N	36	36	-	-	11	11	-
CFBH-T3596	Itabuna	BA	9	control,ND1,ND2	hap219	N	35	36	-	-	2	11	-
CFBH-T3605	Itabuna	BA	9	control,ND1,ND2	hap220	N	-	-	105	105	11	11	-
CFBH-T3609	Itabuna	BA	9	control,ND1,ND2	hap221	N	5	36	-	-	11	11	-
MRT 5806	Jussari	BA	10	control,ND1,ND2	hap268	N	38	35	-	-	2	11	-
MRT 5975	Jussari	BA	10	control,ND1,ND2	hap267	N	38	36	139	105	11	11	16
MTR 13599	Camacan	BA	11	control,ND1,ND2	hap260	N	29	39	134	135	11	11	17
MTR 13608	Camacan	BA	11	control,ND1,ND2	hap261	N	9	9	114	84	11	11	18

					mtDNA		nuclear						
							a.polypep.		crystallin		rhodopsin		STR
number	municipality	state	L	fragments	Hap.	clade	al.1	al.2	al.1	al.2	al.1	al.2	
MTR 13609	Camacan	BA	11	control,ND1,ND2	hap262	N	5	9	-	-	2	11	-
MTR 13610	Camacan	BA	11	control,ND1,ND2	hap261	N	9	39	136	137	11	11	19
MTR 13620	Camacan	BA	11	control,ND1,ND2	hap263	N	-	-	-	-	11	11	-
MTR 13621	Camacan	BA	11	control,ND1,ND2	hap264	N	5	29	-	-	-	-	-
MTR16007	Camacan	BA	11	control,ND1,ND2	hap277	N	35	9	144	119	-	-	-
MTR16011	Camacan	BA	11	control,ND1,ND2	hap278	N	38	9	-	-	-	-	-
MTR16045	Camacan	BA	11	control,ND1,ND2	hap260	N	38	35	105	145	-	-	-
CFBH-T12560	Camacan	BA	11	control,ND1,ND2	hap281	N	5	1	114	147	11	11	20
CFBH-T12561	Camacan	BA	11	control,ND1,ND2	hap282	N	5	35	148	121	11	11	21
CFBH-T12564	Camacan	BA	11	control,ND1,ND2	hap283	N	36	36	-	-	11	11	-
CFBH-T12565	Camacan	BA	11	control,ND1,ND2	hap284	N	5	39	117	149	11	11	22
PUCMG2	Sta. Maria do Salto	MG	12	control,ND1,ND2	hap258	N	5	38	-	-	-	-	-
MNRJ38920	Porto Seguro	BA	13	control,ND1,ND2	hap275	N	38	36	143	105	2	11	23
MNRJ38921	Porto Seguro	BA	13	control,ND1,ND2	hap265	N	46	5	131	117	11	11	24
CFBH-T2389	Grão Mogol	MG	14	control,ND1,ND2	hap207	N	1	1	99	81	11	11	25
PUCMG1	Cristália	MG	15	ND1,ND2	hap279	N	1	47	81	140	-	-	-
MTR 13384	Trancoso	BA	16	control,ND1,ND2	hap255	N	38	38	-	-	2	11	-
MTR 13385	Trancoso	BA	16	control,ND1,ND2	hap256	N	5	43	131	117	-	-	-
MTR 13472	Trancoso	BA	16	control,ND1,ND2	hap257	N	5	38	-	-	11	11	-
MTR 13482	Trancoso	BA	16	control,ND1,ND2	hap258	N	29	44	132	133	11	11	26
MTR 13594	Trancoso	BA	16	control,ND1,ND2	hap259	N	5	44	66	99	11	11	27
MTR 13595	Trancoso	BA	16	control,ND1,ND2	hap230	N	-	-	-	-	2	11	-
MTR 13646	Trancoso	BA	16	control,ND1,ND2	hap265	N	29	35	-	-	11	11	-
CFBH-T4062	Caraíva	BA	17	control,ND1,ND2	hap224	N	38	38	114	115	2	11	28
CFBH-T4063	Caraíva	BA	17	control,ND1,ND2	hap225	N	5	39	116	115	11	11	29
CFBH-T4064	Caraíva	BA	17	control,ND1,ND2	hap226	N	5	38	99	82	11	11	30
CFBH-T4065	Caraíva	BA	17	control,ND1,ND2	hap227	N	35	9	-	-	11	11	-
CFBH-T4066	Caraíva	BA	17	control,ND1,ND2	hap228	N	5	8	-	-	11	11	-
CFBH-T4067	Caraíva	BA	17	control,ND1,ND2	hap229	N	-	-	-	-	11	11	-
CFBH-T4068	Caraíva	BA	17	control,ND1,ND2	hap224	N	-	-	-	-	11	11	-
CFBH-T4069	Caraíva	BA	17	control,ND1,ND2	hap227	N	5	1	114	117	11	8	31
CFBH-T4070	Caraíva	BA	17	control,ND1,ND2	hap230	N	38	38	-	-	11	11	-
CFBH-T4071	Caraíva	BA	17	control,ND1,ND2	hap231	N	38	5	-	-	11	11	-
CFBH-T7689	Prado	BA	18	control,ND1,ND2	hap239	N	5	9	66	127	2	11	32
AF402	Santa Bárbara	MG	19	control,ND1,ND2	hap266	N	20	9	138	121	11	11	33
CFBH-T7652	Nova Lima	MG	20	control,ND1,ND2	hap238	N	1	27	125	125	2	11	-
CFBH-T7660	Nova Lima	MG	20	control,ND1,ND2	hap94	C	-	-	126	126	11	11	-
CFBH-T7664	Nova Lima	MG	20	control,ND1,ND2	hap237	N	5	5	-	-	2	11	34
JC 794	Mariana	MG	21	control,ND1,ND2	hap153	C	8	9	80	81	11	11	35
CFBH-T7482	Ouro Branco	MG	22	control,ND1,ND2	hap92	C	20	8	65	66	2	11	36
CFBH-T7492	Ouro Branco	MG	22	control,ND1,ND2	hap93	C	-	-	67	66	11	11	-
CFBH-T7532	Ouro Branco	MG	22	control,ND1,ND2	hap94	C	5	8	68	69	11	11	37
MNRJ38327	Catas Altas	MG	23	control,ND1,ND2	hap190	C	29	30	87	87	2	2	39
MNRJ38329	Catas Altas	MG	23	ND1,ND2	hap191	C	31	32	88	89	2	11	38
CFBH-T10842	Belo Horizonte	MG	24	control,ND1,ND2	hap116	C	8	9	-	-	7	8	-
MTR 11543	Santa Marta	MG	25	control,ND1,ND2	hap157	C	-	-	83	83	10	10	-
MTR 11548	Santa Marta	MG	25	control,ND1,ND2	hap158	C	-	-	84	85	11	11	-
MTR15751	Espera Feliz	ES	26	control,ND1,ND2	hap193	C	5	5	90	91	2	11	40
MTR15756	Espera Feliz	ES	26	control,ND1,ND2	hap276	N	-	-	-	-	2	11	-
MTR 11588	Córrego Calçado	ES	27	control,ND1,ND2	hap159	C	-	-	84	84	11	11	-
MRT 1252	Ibitirama	ES	28	control,ND1,ND2	hap141	C	1	1	77	77	2	2	41
MRT 1253	Ibitirama	ES	28	control,ND1,ND2	hap142	C	1	24	-	-	2	11	-
MTR 12585	Ibitirama	ES	28	control,ND1,ND2	hap146	C	24	25	78	79	11	11	42
MTR 12609	Ibitirama	ES	28	control,ND1,ND2	hap141	C	-	-	-	-	2	11	-

				mtDNA			nuclear						
							a.polypep.		crystallin		rhodopsin		STR
number	municipality	state	L	fragments	Hap.	clade	al.1	al.2	al.1	al.2	al.1	al.2	
MTR 10732	Caparaó	MG	29	control,ND1,ND2	hap269	N	27	8	82	82	23	23	44
MTR 10733	Caparaó	MG	29	control,ND1,ND2	hap156	C	26	27	82	67	11	11	43
MTR15775	Dores do Rio Preto	ES	30	control,ND1,ND2	hap194	C							-
CFBH-T9177	Sooretama	ES	31	control,ND1,ND2	hap291	N	5	35	97	98	11	11	45
CFBH-T5935	Sooretama	ES	31	control,ND1,ND2	hap233	N	1	1	120	121	-	-	-
CFBH-T5936	Sooretama	ES	31	control,ND1,ND2	hap234	N	5	1	122	123	1	2	47
CFBH-T5945	Sooretama	ES	31	control,ND1,ND2	hap235	N	9	9	114	124	1	2	46
CFBH-T10466	Linhares	ES	32	control,ND1,ND2	hap250	N	5	1	-	-	11	11	-
CFBH-T9043	Linhares	ES	32	control,ND1,ND2	hap242	N	-	-	-	-	11	11	-
CFBH-T9046	Linhares	ES	32	control,ND1,ND2	hap243	N	5	5	129	129	11	11	48
MTR 12132	Linhares	ES	32	control,ND1,ND2	hap270	N	5	1	-	-	11	11	-
MTR 12200	Linhares	ES	32	control,ND1,ND2	hap271	N	5	5	140	131	11	11	49
MTR 12248	Linhares	ES	32	control,ND1,ND2	hap272	N	5	9	99	105	11	11	50
CFBH-T3362	Aracruz	ES	33	control,ND1,ND2	hap208	N	5	1	-	-	11	11	-
CFBH-T3367	Aracruz	ES	33	control,ND1,ND2	hap41	C	1	14	43	43	11	8	51
CFBH-T3370	Aracruz	ES	33	control,ND1,ND2	hap209	N	1	9	-	-	11	11	-
CFBH-T3373	Aracruz	ES	33	control,ND1,ND2	hap210	N	24	9	82	101	11	11	52
CFBH-T9008	Santa Leopoldina	ES	34	control,ND1,ND2	hap240	N	5	1	128	5	11	11	53
CFBH-T9009	Santa Leopoldina	ES	34	control,ND1,ND2	hap240	N	40	41	-	-	19	11	-
CFBH-T9014	Santa Leopoldina	ES	34	control,ND1,ND2	hap241	N	5	1	-	-	11	11	-
CFBH-T9080	Santa Leopoldina	ES	34	control,ND1,ND2	hap110	C	-	-	-	-	2	11	-
CFBH-T9178	Santa Tereza	ES	35	control,ND1,ND2	hap206	N	5	35	99	100	2	11	54
CFBH-T5953	Santa Tereza	ES	35	control,ND1,ND2	hap236	N	-	-	-	-	1	2	-
MNRJ34956	Santa Tereza	ES	35	control,ND1,ND2	hap236	N	24	45	-	-	2	2	-
CFBH-T3393	Vila Velha	ES	36	control,ND1,ND2	hap206	N	-	-	-	-	2	11	-
CFBH-T3397	Vila Velha	ES	36	control,ND1,ND2	hap211	N	24	9	102	103	11	11	55
CFBH-T3400	Vila Velha	ES	36	control	hap212	N							-
CFBH-T3407	Vila Velha	ES	36	control,ND1,ND2	hap213	N	24	24	104	104	2	11	56
LSH1	Guarapari	ES	37	ND1	hap251	N							-
LSH38	Guarapari	ES	37	control,ND1,ND2	hap252	N	5	1	130	100	-	-	-
LSH39	Guarapari	ES	37	control,ND1,ND2	hap253	N	5	5	95	96	2	2	57
LSH60	Anchieta	ES	38	ND1,ND2	hap254	N							-
MTR 1237	S. José do Calçado	ES	39	control,ND1,ND2	hap140	C	5	1	75	76	2	2	58
CFBH-T9324	Teresópolis	RJ	40	control,ND1,ND2	hap204	C	1	1	-	-	2	20	-
CFBH-T9325	Teresópolis	RJ	40	control,ND1,ND2	hap204	C	1	1	-	-	2	20	-
Tfreitas	Itaipava	RJ	41	control,ND1,ND2	hap203	C	1	34	45	45	2	11	59
CFBH-T4380	Petrópolis	RJ	42	control,ND1,ND2	hap68	C	-	-	54	55	11	13	-
CFBH-T9331	Guapimirim	RJ	43	control,ND1,ND2	hap205	C	1	6	4	94	2	13	60
CFBH-T10355	Seropédica	RJ	44	control,ND1,ND2	hap112	C	1	6	-	-	-	-	-
CFBH-T3377	Itaguaí	RJ	45	control,ND1,ND2	hap44	C	-	-	-	-	11	11	-
CFBH-T3381	Itaguaí	RJ	45	control,ND1,ND2	hap208	N	1	6	-	-	2	2	-
CFBH-T3385	Itaguaí	RJ	45	control,ND1,ND2	hap42	C	6	6	-	-	2	2	-
CFBH-T3387	Itaguaí	RJ	45	control,ND1,ND2	hap43	C	-	-	44	45	2	2	-
CFBH-T3422	Itaguaí	RJ	45	control,ND1,ND2	hap44	C	-	-	45	46	2	2	-
CFBH-T2317	Cristina	MG	46	control,ND1,ND2	hap13	C	5	5	6	20	2	2	61
CFBH-T5086	Cristina	MG	46	control,ND1,ND2	hap72	C	5	5	56	57	-	-	-
CFBH-T5087	Cristina	MG	46	control,ND1,ND2	hap73	C	5	5	58	13	2	-	62
CFBH-T3099	S. R. Passa Quatro	SP	47	control,ND1,ND2	hap21	C	5	5	30	59	-	2	-
CFBH-T7699	Bauru	SP	48	control,ND1,ND2	hap95	C	5	5	-	-	2	12	-
CFBH-T7705	Bauru	SP	48	control,ND1,ND2	hap96	C	5	5	13	13	1	2	63
CFBH-T7712	Bauru	SP	48	control,ND1,ND2	hap95	C	5	10	11	20	-	-	-
CFBH-T7717	Bauru	SP	48	control,ND1,ND2	hap95	C	-	-	-	-	10	12	-
CFBH-T7727	Bauru	SP	48	control,ND1,ND2	hap97	C	5	5	70	11	-	-	-
CFBH-T0867	Sto. Ant. do Pinhal	SP	49	control,ND1,ND2	hap5	C	6	6	6	6	2	2	64

				mtDNA			nuclear						
							a.polypep.		crystallin		rhodopsin		STR
number	municipality	state	L	fragments	Hap.	clade	al.1	al.2	al.1	al.2	al.1	al.2	
CFBH-T0868	Sto. Ant. do Pinhal	SP	49	control,ND1,ND2	hap5	C	5	6	7	7	2	2	65
CFBH-T1278	S. Luís Paraitinga	SP	50	control,ND1,ND2	hap8	C	5	5	16	17	2	2	66
CFBH-T6526	S. Luís Paraitinga	SP	50	control,ND1,ND2	hap86	C	5	5	22	6	2	2	67
CFBH-T3170	Nat. da Serra	SP	51	control,ND1,ND2	hap22	C	-	-	6	4	2	2	-
CFBH-T3174	Nat. da Serra	SP	51	control,ND1,ND2	hap4	C	5	5	32	25	2	2	68
CFBH-T0336	Ubatuba	SP	52	control,ND1,ND2	hap4	C	5	5	4	5	2	2	69
CFBH-T2588	Ubatuba	SP	52	control,ND1,ND2	hap16	C	5	6	24	25	2	11	70
CFBH-T3628	Ubatuba	SP	52	control,ND1,ND2	hap47	C	14	15	4	5	2	2	71
CFBH-T3633	Ubatuba	SP	52	control,ND1,ND2	hap48	C	6	6	45	22	2	2	72
CFBH-T3638	Ubatuba	SP	52	control,ND1,ND2	hap49	C	6	6	49	49	2	2	73
CFBH-T3659	Ubatuba	SP	52	control,ND1,ND2	hap50	C	6	6	49	49	2	2	74
CFBH-T3803	Caraguatatuba	SP	53	control,ND1,ND2	hap53	C	5	16	22	22	2	2	75
CFBH-T3806	Caraguatatuba	SP	53	control,ND1,ND2	hap13	C	5	6	41	9	2	2	76
CFBH-T3834	Caraguatatuba	SP	53	control,ND1,ND2	hap54	C	5	5	13	28	2	2	77
CFBH-T3836	Caraguatatuba	SP	53	control,ND1,ND2	hap55	C	5	6	22	41	2	2	78
CFBH-T8867	São Sebastião	SP	54	ND1,ND2	hap107	C	5	17	-	-	2	2	-
CFBH-T8890	São Sebastião	SP	54	ND1,ND2	hap108	C	5	6	-	-	1	2	-
CFBH-T8891	São Sebastião	SP	54	control,ND1,ND2	hap109	C	5	5	-	-	2	2	-
CFBH-T8900	São Sebastião	SP	54	control,ND1,ND2	hap109	C	5	6	6	22	2	2	79
CFBH-T2664	Ilha Bela	SP	55	control,ND1,ND2	hap17	C	5	6	22	22	2	2	80
CFBH-T4008	Ilha Bela	SP	55	control,ND1,ND2	hap63	C	5	5	-	-	2	2	-
CFBH-T4009	Ilha Bela	SP	55	control,ND1,ND2	hap64	C	5	6	-	-	2	2	-
CFBH-T4010	Ilha Bela	SP	55	control,ND1,ND2	hap65	C	5	5	22	22	2	2	81
CFBH-T6033	Ilha Bela	SP	55	control,ND1,ND2	hap17	C	5	5	22	22	2	2	82
CFBH-T6048	Ilha Bela	SP	55	control,ND1,ND2	hap63	C	5	6	-	-	2	2	-
CFBH-T6053	Ilha Bela	SP	55	control,ND1,ND2	hap85	C	6	6	22	22	2	2	83
CFBH-T6054	Ilha Bela	SP	55	control,ND1,ND2	hap13	C	5	5	22	22	2	2	84
H-466	Bertioga	SP	56	control,ND1,ND2	hap185	C	6	6	5	9	-	-	-
H-478	Bertioga	SP	56	control,ND1,ND2	hap183	C	5	17	-	-	2	2	-
H-485	Bertioga	SP	56	control,ND1,ND2	hap109	C	5	5	-	-	2	2	-
H-488	Bertioga	SP	56	control,ND1,ND2	hap184	C	5	5	6	22	-	-	-
UF94/18	Bertioga	SP	56	control,ND1,ND2	hap109	C	6	6	-	-	2	2	-
UF94/30	Bertioga	SP	56	control,ND1,ND2	hap183	C	5	12	22	22	2	2	85
CFBH-T1956	Cubatão	SP	57	control,ND1,ND2	hap10	C	5	10	6	19	2	2	86
CFBH-T2516	Cubatão	SP	57	control,ND1,ND2	hap14	C	5	5	21	22	10	2	87
CFBH-T2517	Cubatão	SP	57	control,ND1,ND2	hap15	C	5	5	23	22	2	2	88
CFBH-T3491	Cubatão	SP	57	control,ND1,ND2	hap45	C	-	-	47	9	2	2	-
CFBH-T3896	Cubatão	SP	57	control,ND1,ND2	hap57	C	5	5	-	-	2	2	-
CFBH-T3897	Cubatão	SP	57	control,ND1,ND2	hap58	C	5	5	23	41	5	2	89
CFBH-T3910	Cubatão	SP	57	control,ND1,ND2	hap59	C	5	17	-	-	2	2	-
CFBH-T5522	Cubatão	SP	57	control,ND1,ND2	hap75	C	5	1	63	37	2	2	90
AF1430	Santos	SP	58	control,ND1,ND2	hap179	C	5	1	22	22	2	2	93
AF1435	Santos	SP	58	control,ND1,ND2	hap180	C	5	17	-	-	2	2	-
AF1443	Santos	SP	58	control,ND1,ND2	hap181	C	5	5	6	22	12	19	94
CFBH-T3953	Santos	SP	58	control,ND1,ND2	hap61	C	5	18	22	41	2	2	91
CFBH-T3954	Santos	SP	58	control,ND1,ND2	hap62	C	5	5	41	22	2	2	92
MTR 16675	Santo André	SP	59	control,ND1,ND2	hap13	C	5	5	46	22	2	2	95
MTR10400	Santo André	SP	59	control,ND1,ND2	hap154	C	5	10	35	22	-	-	-
MTR10402	Santo André	SP	59	control,ND1,ND2	hap155	C	5	5	-	-	2	2	-
IT-H0023	São Bernardo	SP	60	control,ND1,ND2	hap163	C	10	10	22	22	2	2	96
IT-H0025	São Bernardo	SP	60	control,ND1,ND2	hap164	C	5	10	22	22	2	2	97
CFBH-T10514	Itanhaém	SP	61	control,ND1,ND2	hap113	C	5	5	11	12	2	2	100
CFBH-T3856	Itanhaém	SP	61	control,ND1,ND2	hap56	C	5	5	52	53	2	2	98
CFBH-T3912	Itanhaém	SP	61	control,ND1,ND2	hap60	C	5	10	20	35	2	2	99

				mtDNA			nuclear						
							a.polypep.		crystallin		rhodopsin		STR
number	municipality	state	L	fragments	Hap.	clade	al.1	al.2	al.1	al.2	al.1	al.2	
CFBH-T5869	Peruíbe	SP	62	control,ND1,ND2	hap83	C							-
CFBH-T5870	Peruíbe	SP	62	control,ND1,ND2	hap83	C	5	5	26	29	2	2	101
CFBH-T5871	Peruíbe	SP	62	control,ND1,ND2	hap84	C	4	5	28	8	2	2	102
AF122	Iguape	SP	63	control,ND1,ND2	hap143	C	4	16	-	-	2	2	-
AF147	Iguape	SP	63	control,ND1,ND2	hap144	C	5	5	-	-	2	2	-
AF151	Iguape	SP	63	control,ND1,ND2	hap145	C	5	5	11	11	2	2	106
AF160	Iguape	SP	63	control,ND1,ND2	hap147	C	5	16	9	9	2	2	107
AF169	Iguape	SP	63	control,ND1,ND2	hap90	C	5	5	9	11	-	-	-
CFBH-T2246	Iguape	SP	63	control,ND1,ND2	hap11	C	5	5	-	-	9	9	-
CFBH-T2248	Iguape	SP	63	control,ND1,ND2	hap12	C	5	5	-	-	2	2	-
CFBH-T4031	Iguape	SP	63	control,ND1,ND2	hap66	C	5	5	8	20	2	2	103
CFBH-T4032	Iguape	SP	63	control,ND1,ND2	hap67	C	5	1	20	11	2	2	104
CFBH-T5144	Iguape	SP	63	control,ND1,ND2	hap74	C	1	16	60	61	2	2	105
CFBH-T6957	Iguape	SP	63	control,ND1,ND2	hap88	C	19	19	-	-	2	2	-
CFBH-T6976	Iguape	SP	63	control,ND1,ND2	hap89	C	5	5	-	-	2	2	-
CFBH-T7034	Iguape	SP	63	control,ND1,ND2	hap90	C	5	5	9	27	-	-	-
CFBH-T6952	Pariquera Açú	SP	64	control,ND1,ND2	hap87	C	10	16	-	-	2	11	-
CFBH-T7322	Ilha Comprida	SP	65	control,ND1,ND2	hap91	C	5	5	13	13	2	2	108
CFBH-T10697	Cananéia	SP	66	control,ND1,ND2	hap103	C	5	5	13	13	2	2	111
CFBH-T8433	Cananéia	SP	66	control,ND1,ND2	hap103	C	5	5	-	-	2	2	-
CFBH-T8434	Cananéia	SP	66	control,ND1,ND2	hap104	C	5	5	13	11	1	2	109
CFBH-T8547	Cananéia	SP	66	control,ND1,ND2	hap105	C	5	23	13	13	2	2	110
CFBH-T8548	Cananéia	SP	66	control,ND1,ND2	hap104	C	5	5	-	-	2	2	-
CFBH-T8683	Cananéia	SP	66	control,ND1,ND2	hap106	C	5	5	-	-	2	2	-
CFBH-T5155	Ilha do Cardoso	SP	67	control,ND1,ND2	hap32	C	5	5	62	22	2	2	112
AF403	São Paulo	SP	68	control	hap149	C	5	10	20	22	2	2	113
MTR 9957	São Paulo	SP	68	control,ND1,ND2	hap113	C	5	5	41	22	2	2	115
MTR 9958	São Paulo	SP	68	control,ND1,ND2	hap188	C	5	5	-	-	2	2	-
MTR 9959	São Paulo	SP	68	control,ND1,ND2	hap189	C	5	5	41	41	2	2	116
MTR 9960	São Paulo	SP	68	control,ND1,ND2	hap5	C	4	5	-	-	2	2	-
MTR 9963	São Paulo	SP	68	control,ND1,ND2	hap182	C	5	5	6	11	2	2	114
CFBH-T3108	Cotia	SP	69	control,ND1,ND2	hap13	C	5	5	28	32	-	-	-
CFBH-T8274	Biritiba Mirim	SP	70	control,ND1,ND2	hap29	C	5	5	6	9	-	-	-
CFBH-T8275	Biritiba Mirim	SP	70	control,ND1,ND2	hap101	C	17	17	14	26	2	2	117
CFBH-T8276	Biritiba Mirim	SP	70	control,ND1,ND2	hap102	C	5	5	-	-	-	-	-
Uniban2555	Biritiba Mirim	SP	70	control,ND1,ND2	hap5	C	28	17	6	6	2	11	118
CFBH-T3180	Santa Isabel	SP	71	control,ND1,ND2	hap5	C	5	6	33	22	2	2	119
CFBH-T3184	Santa Isabel	SP	71	control,ND1,ND2	hap23	C	5	5	22	4	2	2	120
CFBH-T3189	Santa Isabel	SP	71	control,ND1,ND2	hap24	C	5	5	6	6	-	-	-
CFBH-T3190	Santa Isabel	SP	71	control,ND1,ND2	hap24	C	5	5	20	6	12	6	124
CFBH-T3194	Santa Isabel	SP	71	control,ND1,ND2	hap25	C	5	11	4	22	-	-	-
CFBH-T3195	Santa Isabel	SP	71	control,ND1,ND2	hap26	C	5	5	34	22	2	2	121
CFBH-T3202	Santa Isabel	SP	71	control,ND1,ND2	hap27	C	-	-	35	22	2	2	-
CFBH-T3204	Santa Isabel	SP	71	control,ND1,ND2	hap28	C	12	11	36	37	-	-	-
CFBH-T3206	Santa Isabel	SP	71	control,ND1,ND2	hap29	C	6	6	6	6	1	2	122
CFBH-T4426	Santa Isabel	SP	71	control,ND1,ND2	hap69	C	5	5	35	9	14	2	123
CFBH-T4427	Santa Isabel	SP	71	control,ND1,ND2	hap70	C	5	5	41	11	-	-	-
MTR15559	Iperó	SP	72	ND1,ND2	hap192	C	5	5	-	-	12	12	-
IT-H0437	Juquitiba	SP	73	control,ND1,ND2	hap167	C	5	5	-	-	12	6	-
IT-H0438	Juquitiba	SP	73	control,ND1,ND2	hap168	C	5	10	22	11	2	18	125
IT-H0439	Juquitiba	SP	73	control,ND1,ND2	hap45	C	5	10	-	-	-	-	-
IT-H0459	Juquitiba	SP	73	control,ND1,ND2	hap169	C	5	5	-	-	2	2	-
IT-H0461	Juquitiba	SP	73	control,ND1,ND2	hap66	C	17	10	20	11	-	-	-
IT-H0462	Juquitiba	SP	73	control,ND1,ND2	hap113	C	5	5	35	35	1	2	127

				mtDNA			nuclear						
							a.polypep.		crystallin		rhodopsin		STR
number	municipality	state	L	fragments	Hap.	clade	al.1	al.2	al.1	al.2	al.1	al.2	
IT-H0463	Juquitiba	SP	73	control,ND1,ND2	hap45	C	5	5	6	41	12	12	128
IT-H0468	Juquitiba	SP	73	control,ND1,ND2	hap170	C	5	5	9	11	2	2	126
IT-H0538	Juquitiba	SP	73	ND1,ND2	hap171	C	5	5	-	-	-	-	-
AF303	Carapicuíba	SP	74	control,ND1,ND2	hap45	C	5	5	9	11	6	6	129
AF304	Carapicuíba	SP	74	control,ND1,ND2	hap5	C	5	5	-	-	-	-	-
AF316	Carapicuíba	SP	74	control,ND2	hap148	C	5	5	20	20	-	-	-
AF423	Carapicuíba	SP	74	control,ND2	hap150	C	5	17	34	41	-	-	-
ALC86/77	Piedade	SP	75	control,ND1,ND2	hap5	C	5	5	28	28	1	12	130
ALC86/80	Piedade	SP	75	control,ND1,ND2	hap113	C	5	17	-	-	2	2	-
ALC86/81	Piedade	SP	75	control,ND1,ND2	hap113	C	5	5	41	41	12	12	131
ALC87/1	Piedade	SP	75	control,ND1,ND2	hap160	C	5	5	28	28	10	2	132
ALC87/3	Piedade	SP	75	control,ND1,ND2	hap161	C	5	5	-	-	-	-	-
ALC87/5	Piedade	SP	75	control,ND1,ND2	hap162	C	5	5	-	-	2	2	-
H023	Piedade	SP	75	control,ND1,ND2	hap113	C	5	5	64	20	12	6	135
H035	Piedade	SP	75	control,ND1,ND2	hap186	C	5	17	41	22	-	-	-
IT-H0207	Piedade	SP	75	control,ND1,ND2	hap165	C	5	5	6	41	12	12	133
IT-H0208	Piedade	SP	75	control,ND1,ND2	hap166	C	5	5	-	-	2	2	-
IT-H0209	Piedade	SP	75	control,ND1,ND2	hap66	C	17	10	20	11	-	-	-
UF88/60	Piedade	SP	75	control,ND1,ND2	hap113	C	5	5	64	20	12	6	134
H0300	Buri	SP	76	control,ND1,ND2	hap174	C	5	5	-	-	10	1	-
H0355	Buri	SP	76	control,ND1,ND2	hap151	C	5	5	20	29	1	1	141
H0356	Buri	SP	76	control,ND1,ND2	hap187	C	5	5	10	11	1	1	140
H0366	Buri	SP	76	control,ND1,ND2	hap152	C	5	5	20	11	10	1	139
IT-H0573	Buri	SP	76	control,ND1,ND2	hap173	C	5	5	29	29	1	12	137
IT-H0580	Buri	SP	76	control,ND1,ND2	hap80	C	5	10	-	-	10	10	-
IT-H0581	Buri	SP	76	control,ND1,ND2	hap174	C	5	5	-	-	10	1	-
IT-H0583	Buri	SP	76	control,ND1,ND2	hap175	C	10	10	10	26	1	1	138
IT-H0610	Buri	SP	76	control,ND1,ND2	hap151	C	5	10	9	11	-	-	-
IT-H0612	Buri	SP	76	control,ND1,ND2	hap152	C	5	5	26	27	12	12	136
IT-H0613	Buri	SP	76	control,ND1,ND2	hap151	C	5	5	-	-	1	1	-
CFBH-T5679	Ribeirão Grande	SP	77	control,ND1,ND2	hap76	C	5	5	11	29	10	1	142
CFBH-T5680	Ribeirão Grande	SP	77	control,ND1,ND2	hap77	C	5	5	64	9	1	12	143
CFBH-T5681	Ribeirão Grande	SP	77	control,ND1,ND2	hap78	C	5	10	28	11	12	12	144
CFBH-T5682	Ribeirão Grande	SP	77	control,ND1,ND2	hap79	C	5	5	-	-	10	12	-
CFBH-T5683	Ribeirão Grande	SP	77	control,ND1,ND2	hap80	C	5	5	-	-	1	12	-
CFBH-T5684	Ribeirão Grande	SP	77	control,ND1,ND2	hap81	C	5	10	-	-	1	12	-
CFBH-T5685	Ribeirão Grande	SP	77	control,ND1,ND2	hap82	C	5	5	26	26	1	12	145
CFBH-T5686	Ribeirão Grande	SP	77	control,ND1,ND2	hap79	C	5	10	-	-	2	12	-
CFBH-T5687	Ribeirão Grande	SP	77	control,ND1,ND2	hap80	C	5	5	29	9	1	2	146
CFBH-T5688	Ribeirão Grande	SP	77	control,ND1,ND2	hap80	C	5	5	-	-	1	12	-
CFBH-T5041	Guapiara	SP	78	control,ND1,ND2	hap71	C	5	10	40	11	1	12	147
CFBH-T1034	Ribeirão Branco	SP	79	control,ND1,ND2	hap6	C	4	5	10	10	-	-	-
CFBH-T1035	Ribeirão Branco	SP	79	control,ND1,ND2	hap7	C	5	5	9	10	1	2	148
IIH-149	Wenceslau Brás	PR	80	control,ND1,ND2	hap176	C	5	1	10	26	12	12	149
IIH-172	Wenceslau Brás	PR	80	control,ND1,ND2	hap177	C	5	5	10	26	10	12	150
IIH-224	Wenceslau Brás	PR	80	control,ND1,ND2	hap178	C	5	5	10	10	10	12	151
II - H046	Pinhalão	PR	81	control,ND1,ND2	hap172	C	5	1	10	26	10	12	152
II - H114	Ortigueira	PR	82	control,ND1,ND2	hap151	C	5	5	86	13	-	-	-
CFBH-T11188	Ortigueira	PR	82	control,ND1,ND2	hap121	C	5	1	9	9	12	12	153
CFBH-T9476	Teodoro Sampaio	SP	83	control,ND1,ND2	hap199	C	5	1	10	26	12	12	154
CFBH-T7881	Teodoro Sampaio	SP	83	control,ND1,ND2	hap200	C	5	1	93	26	10	12	155
CFBH-T7897	Teodoro Sampaio	SP	83	control,ND1,ND2	hap201	C	5	5	10	26	10	12	156
CFBH-T7898	Teodoro Sampaio	SP	83	control,ND1,ND2	hap201	C	5	1	-	-	10	12	-
CFBH-T9512	Teodoro Sampaio	SP	83	control,ND1,ND2	hap202	C	1	1	10	26	10	10	157

				mtDNA			nuclear						
							a.polypep.		crystallin		rhodopsin		STR
number	municipality	state	L	fragments	Hap.	clade	al.1	al.2	al.1	al.2	al.1	al.2	
CFBH-T7899	Teodoro Sampaio	SP	83	control,ND1,ND2	hap202	C	5	1	-	-	10	10	-
CFBH-T9513	Teodoro Sampaio	SP	83	control,ND1,ND2	hap200	C	5	5	-	-	10	12	-
CFBH-T9514	Teodoro Sampaio	SP	83	control,ND1,ND2	hap200	C	5	5	10	26	10	12	158
IIBPH1333	Canindeyú	PAR	84	control,ND1,ND2	hap119	C	5	1	10	9	10	12	159
IIBPH1173	Itapúa	PAR	85	control,ND1,ND2	hap117	C	5	5	9	9	12	12	160
IIBPH1191	Itapúa	PAR	85	control,ND1,ND2	hap118	C	5	5	9	9	10	12	161
IIBPH1336	Itapúa	PAR	85	ND1	hap120	C	-	-	9	9	10	12	-
IIBPH1342	Itapúa	PAR	85	control,ND1,ND2	hap121	C	5	1	9	9	12	12	162
IIBPH1353	Itapúa	PAR	85	control,ND1,ND2	hap113	C	5	5	20	9	10	12	163
IIBPH1356	Itapúa	PAR	85	control,ND1,ND2	hap113	C	5	5	9	9	10	10	164
MVS243	Adrianópolis	PR	86	control,ND1,ND2	hap196	c1	4	5	28	13	-	-	-
MVS244	Adrianópolis	PR	86	control,ND1,ND2	hap197	C	33	33	92	26	10	12	165
MVS242	Ribeira	SP	87	control,ND1,ND2	hap195	C	4	5	28	13	-	-	-
CFBH-T3619	Quatro Barras	PR	88	control,ND1,ND2	hap46	c1	-	-	48	11	5	6	-
CFBH-T9193	Quatro Barras	PR	88	control,ND1,ND2	hap124	c1	4	4	41	41	6	17	166
CFBH-T12598	Antonina	PR	89	control,ND1,ND2	hap3	c1	4	5	-	-	1	2	-
CFBH-T3084	Antonina	PR	89	control,ND1,ND2	hap18	C	5	5	26	27	-	-	-
CFBH-T3085	Antonina	PR	89	control,ND1,ND2	hap19	c1	4	7	28	26	10	1	167
CFBH-T3086	Antonina	PR	89	control,ND1,ND2	hap20	C	-	-	29	27	1	12	-
CFBH-T3304	Morretes	PR	90	control,ND1,ND2	hap30	c1	5	7	11	38	-	-	-
CFBH-T3306	Morretes	PR	90	control,ND1,ND2	hap31	c1	4	5	11	9	2	2	168
CFBH-T3310	Morretes	PR	90	control,ND1,ND2	hap32	C	5	1	27	11	1	12	169
CFBH-T3312	Morretes	PR	90	control,ND1,ND2	hap33	c1							-
K970	São José dos Pinhais	PR	91	control,ND1,ND2	hap123	c1	5	1	-	-	10	2	-
CFBH-T10261	Guaratuba	PR	92	control,ND1,ND2	hap111	c1	4	7	8	9	-	-	-
CFBH-T3665	Guaratuba	PR	92	control,ND1,ND2	hap51	c1	4	5	8	11	-	-	-
CFBH-T3666	Guaratuba	PR	92	control,ND1,ND2	hap51	c1	4	5	8	11	-	-	-
CFBH-T3672	Guaratuba	PR	92	control,ND1,ND2	hap52	c1	-	-	50	51	-	-	-
CFBH-T3679	Guaratuba	PR	92	control,ND1,ND2	hap33	c1	5	7	22	11	-	-	-
CFBH-T3314	Itapoá	SC	93	control,ND1,ND2	hap34	c1	5	5	27	26	1	2	170
CFBH-T9201	Massaranduba	SC	94	control,ND1,ND2	hap131	c1	5	7	9	11	-	-	-
CFBH-T9202	Massaranduba	SC	94	control,ND1,ND2	hap132	c1	4	1	-	-	1	5	-
CFBH-T9202	Massaranduba	SC	94	control,ND1,ND2	hap133	c1	4	5	9	9	-	-	-
CFBH-T9204	Massaranduba	SC	94	control,ND1,ND2	hap134	c1	7	7	74	9	1	1	171
CFBH-T9205	Massaranduba	SC	94	control,ND1,ND2	hap135	c1	1	7	73	9	5	6	172
CFBH-T9206	Massaranduba	SC	94	control,ND1,ND2	hap136	c1	4	4	38	41	10	12	173
CFBH-T9207	Massaranduba	SC	94	control,ND1,ND2	hap134	c1	7	7	-	-	5	5	-
CFBH-T9208	Massaranduba	SC	94	control,ND1,ND2	hap136	c1	1	7	9	11	-	-	-
CFBH-T9209	Massaranduba	SC	94	control,ND1,ND2	hap137	c1	4	1	26	73	12	6	174
CFBH-T9210	Massaranduba	SC	94	control,ND1,ND2	hap138	c1	7	7	-	-	5	5	-
CFBH-T9211	Massaranduba	SC	94	control,ND1,ND2	hap139	c1	7	7	15	9	5	5	175
CFBH-T3316	Rio dos Cedros	SC	95	control,ND1,ND2	hap35	c1	-	-	39	11	1	1	-
CFBH-T3320	Rio dos Cedros	SC	95	control,ND1,ND2	hap36	c1	-	-	40	41	1	5	-
CFBH-T3323	Rio dos Cedros	SC	95	control,ND1,ND2	hap37	c1	4	4	42	11	1	12	176
CFBH-T3343	Blumenau	SC	96	control,ND1,ND2	hap38	c1	-	-	14	9	-	-	-
CFBH-T3348	Blumenau	SC	96	control,ND1,ND2	hap39	c1	4	4	11	15	-	-	-
CFBH-T3351	Blumenau	SC	96	control,ND1,ND2	hap40	c1	13	7	15	9	1	5	177
CFBH-T9194	Blumenau	SC	96	control,ND1,ND2	hap125	c1	4	7	15	15	6	6	178
CFBH-T9195	Blumenau	SC	96	control,ND1,ND2	hap126	c1	4	1	72	11	1	1	179
CFBH-T9196	Blumenau	SC	96	control,ND1,ND2	hap126	c1	4	7	15	9	6	6	180
CFBH-T9197	Blumenau	SC	96	control,ND1,ND2	hap127	c1	4	1	73	9	-	-	-
CFBH-T9198	Blumenau	SC	96	control,ND1,ND2	hap128	c1	7	7	15	73	10	5	181
CFBH-T9199	Blumenau	SC	96	control,ND1,ND2	hap129	c1	1	7	9	72	1	1	182
CFBH-T9200	Blumenau	SC	96	control,ND1,ND2	hap130	c1	4	7	26	41	-	-	-

				mtDNA			nuclear						
							a.polypep.		crystallin		rhodopsin		STR
number	municipality	state	L	fragments	Hap.	clade	al.1	al.2	al.1	al.2	al.1	al.2	
CFBH-T10731	Gov. Celso Ramos	SC	97	control,ND1,ND2	hap114	c1	4	1	11	14	5	6	183
CFBH-T10732	Gov. Celso Ramos	SC	97	control,ND1,ND2	hap115	c1	1	1	11	15	1	2	184
CFBH-T1735	Itapema	SC	98	control,ND1,ND2	hap9	c1	4	4	18	11	-	-	-
PUCRS	S.. Am. Imperatriz	SC	99	control,ND1,ND2	hap198	c1	4	1	26	11	12	6	185
CFBH-T8092	Anitápolis	SC	100	control,ND1,ND2	hap98	c1	21	22	-	-	3	15	-
CFBH-T8093	Anitápolis	SC	100	control,ND1,ND2	hap295	S	-	-	154	155	3	25	-
CFBH-T8094	Anitápolis	SC	100	control,ND1	hap99	c1	1	1	11	11	-	-	-
CFBH-T8095	Anitápolis	SC	100	control,ND1,ND2	hap100	c1	-	-	-	-	-	-	-
CFBH-T8096	Anitápolis	SC	100	control,ND1,ND2	hap100	c1	22	22	155	155	25	16	-
CFBH-T8097	Anitápolis	SC	100	control,ND1,ND2	hap100	c1	1	1	-	-	6	16	-
CFBH-T8098	Anitápolis	SC	100	control,ND1,ND2	hap296	S	1	1	71	11	-	-	193
CFBH-T8099	Anitápolis	SC	100	control,ND1,ND2	hap297	S	-	-	155	155	3	3	-
ELMG453	Ponte Serrada	SC	101	control,ND1,ND2	hap299	S							-
CFBH-T11195	Ponte Serrada	SC	101	control,ND1,ND2	hap122	C	5	5	28	28	12	12	186
CFBH-T11196	Fernandes Pinheiro	PR	102	control,ND1,ND2	hap292	S	21	22	159	160	3	3	187
MVS398	Salete	SC	103	ND1,ND2	hap304	S	-	-	-	-	10	5	-
CFBH-T10793	Xavantina	SC	104	control,ND1,ND2	hap293	S	51	52	151	151	1	2	189
CFBH-T10794	Xavantina	SC	104	control,ND1,ND2	hap294	S	53	54	152	153	2	2	190
CFBH-T9184	Xavantina	SC	104	control,ND1,ND2	hap299	S	-	-	156	156	3	15	-
CFBH-T9186	Xavantina	SC	104	control,ND1,ND2	hap299	S	53	56	156	156	3	4	188
CFBH-T9188	Xavantina	SC	104	control,ND1,ND2	hap299	S	-	-	155	156	3	25	-
EMLG234	Guatambú	SC	105	control,ND1,ND2	hap299	S	57	56	156	155	-	-	-
EMLG235	Guatambú	SC	105	control,ND1,ND2	hap299	S	21	22	157	5	-	-	-
EMLG236	Guatambú	SC	105	control,ND1,ND2	hap300	S	-	-	158	5	-	-	-
EMLG237	Guatambú	SC	105	control,ND1,ND2	hap299	S							-
EMLG253	Guatambú	SC	105	control,ND1,ND2	hap299	S	58	59	156	156	25	25	191
EMLG453	Guatambú	SC	105	-	-	-	21	22	-	-	3	25	-
flona062007	Guatambú	SC	105	control,ND1,ND2	hap292	S	-	-	159	160	3	3	-
flona1603	Guatambú	SC	105	control,ND1,ND2	hap292	S	21	21	156	156	3	25	192
MNRJ33006	Mato Castelhano	RS	106	control,ND1,ND2	hap302	S	13	22	162	163	3	25	194
MNRJ33012	Mato Castelhano	RS	106	control,ND1,ND2	hap303	S	61	22	163	163	25	25	195
CFBH-T8298	Cotiporã	RS	107	control,ND1,ND2	hap298	S	55	55	-	-	25	25	-
UFRGST667	Nova Roma do Sul	RS	108	control,ND1,ND2	hap305	S	-	-	163	163	25	25	-
CFBH-T9213	Bento Gonçalves	RS	109	control,ND1,ND2	hap301	S	21	60	155	161	3	15	196
	outgroups												
CFBH-T1338	*R. arenarum*			control,ND1,ND2									
CFBH-T3051	*R. icterica*			control,ND1,ND2									
CFBH-T3354	*R. icterica*			control,ND1									
CFBH-T3361	*R. granulosa*			control,ND1									
CFBH-T5092	*R. merianae*			control,ND1,ND2									
CFBH-T5731	*R.merianae*			control,ND1,ND2									
CFBH-T8885	*R. icterica*			control,ND1									
CFBH-T8933	*R. icterica*			ND1									
